# Comparison of short-segment and long-segment fixation in treatment of degenerative scoliosis and analysis of factors associated with adjacent spondylolisthesis

**DOI:** 10.1515/med-2024-0983

**Published:** 2024-06-21

**Authors:** Long Pang, Zhihui Gao, Long Ma, Yaping Li, Zhidong Lu, Liang Zhang, Peng Li, Long Wu

**Affiliations:** Third Orthopedic Department, General Hospital of Ningxia Medical University, Yinchuan, 750004, China

**Keywords:** long-segment internal fixation, short-segment internal fixation, degenerative scoliosis, adjacent vertebral disease, coronal Cobb’s angle

## Abstract

The bleeding time and amount in the short-segment group were shorter than in the long-segment group, and the bleeding volume was less than in the long-segment group. The Japanese Orthopaedic Association low back pain score, Oswestry Dysfunction Index, and lumbar spine stiffness disability index score of the two groups were significantly improved preoperatively, postoperatively, and at 6 months, 1 year, and 2 years post-operation. The differences were statistically significant at different time points within the groups. Neurological function improved to varying degrees postoperatively. The Cobb angle was significantly higher in both groups (*P* < 0.05). Adjacent vertebral disease occurred in 10 of 64 patients with short-segment fixation, with a prevalence of 15.6%. Preoperative pelvic tilt angle, preoperative pelvic projection angle (PPA), preoperative degree of matching of PPA to LL (PI-LL), and preoperative coronal Cobb angle were higher in patients with adjacent vertebral disease. There were varying degrees of improvement in low back pain and spinal function after short-segment decompression and fusion internal fixation. However, the patients are generally elderly and at risk of persistent low back pain and accelerated degeneration of adjacent segments.

## 1 Introduction

In recent years, with the aging population in China, the incidence of adult degenerative scoliosis (ADS) has significantly increased and continues to rise with age [1]. As the quality of life improves, more adult patients with degenerative scoliosis (DS) are opting for surgical intervention when conservative treatments fail to yield satisfactory results. Consequently, medical staff are increasingly concerned with the growing number of patients with DS seeking surgical solutions.

ADS refers to a spinal deformity that develops after skeletal maturity without a prior history of scoliosis. It is caused by the progressive degeneration of intervertebral discs, vertebral bodies, and intervertebral joints, characterized by a Cobb angle >10° on X-rays. ADS mainly occurs in the lumbar and thoracolumbar spines and is prevalent in individuals over 55 years old [2]. Patients often present with symptoms such as lower limb radiating pain, low back pain, and trunk imbalance. Studies on the pathological mechanism of ADS have found that lateral curvature, combined with spinal canal stenosis and lateral spondylolisthesis of the vertebral body, causes neurogenic claudication [3]. The nerve root on the concave side is compressed while the convex side is stretched, leading to neurological symptoms. Low back pain is primarily due to lumbar facet joint disease and intervertebral disc degeneration. Sagittal plane spine imbalance can cause lumbosacral muscle strain, further contributing to low back pain [4].

Previous studies categorized scoliosis as an abnormal transverse curvature deformity of the spine. However, with advancements in imaging technology and a deep understanding of biomechanics and spinal anatomy, it is now recognized as a three-dimensional deformity [5]. In conservative treatment, patients are typically prescribed muscle relaxants and non-steroidal anti-inflammatory drugs to manage pain [6]. If symptoms persist, treatment options such as epidural steroid injections, articular injections, nerve root blocks, and oral analgesics may be considered. Physical therapy and aquatic exercise therapy can complement drug therapy, promoting dysfunction relief and maintaining muscle tension [7]. While the efficacy of bracing in preventing lateral bending progression remains uncertain, theoretically, it can provide pain relief through its immobilizing effect. However, prolonged brace wear may lead to disuse atrophy or motor dysfunction in relevant muscles. Unfortunately, some patients experience worsening symptoms such as low back and leg pain and nerve root pain despite conservative treatment, significantly impacting their daily lives. Surgical intervention becomes necessary for patients who do not respond effectively to conservative measures.

Surgical treatment is a common method for alleviating the symptoms of patients, providing pain relief, stabilizing the spine, and correcting spinal deformities to a certain extent. In the surgical treatment of DS, the primary approach involves internal fixation, fusion, and decompression of the segment responsible for symptoms. Long- and short-segment internal fixation and fusion are commonly employed techniques. Short-segment internal fixation and fusion involve fixing the fusion segment shorter than the adjacent upper and lower vertebrae of the affected vertebral body. This typically includes one vertebra above and below the fractured vertebra. In contrast, long-segment internal fixation and fusion extend to or beyond the upper and lower vertebrae of the affected vertebral body, with fixation spanning two or more vertebrae above and below the injured segment. Researchers both domestically and internationally have found that long-segment internal fixation and fusion effectively achieve fixation, thereby enhancing spinal balance [8]. Short-segment internal fixation and fusion can stabilize the spine and alleviate symptoms when the fusion segment is short. However, there remains considerable controversy among experts and scholars worldwide regarding the preferred method of fixed treatment for DS.

Patients with DS often opt for surgical intervention primarily due to symptoms associated with lumbar stenosis, typically involving shorter spinal segments than scoliosis cases. While long-segment spinal fusion offers greater stability, it comes with drawbacks such as reduced active segments, limited spinal range of motion, increased costs, and a higher risk of perioperative complications [8]. Considering that older patients frequently present with multiple medical comorbidities, symptom relief can often be achieved through short-segment decompression fusion procedures targeting the upper and lower vertebral boundaries of the scoliosis region [9].

With an aging population, adjacent spondylosis (ASD) arises as a common complication following lumbar fusion surgery, characterized by degenerative changes in segments adjacent to the fused region, which can manifest as imaging or symptomatic ASD [10]. In this study, ASD is defined as a pathological process resulting from disc degeneration, leading to clinical symptoms such as neuroinfarct lesions, stenosis, and instability. ASD represents the most prevalent complication of short-segment fusion for DS and significantly influences reoperation rates post-fusion. Despite widespread reporting on the incidence and factors associated with ASD, inconsistencies such as varying fusion techniques and preoperative diagnoses hinder definitive conclusions. The factors contributing to ASD development remain controversial, with limited research available. Therefore, this study aims to assess the efficacy of short-segment decompression fusion for DS and explore potential factors associated with the occurrence of ASD. By doing so, it seeks to offer insights for optimizing clinical treatment planning and enhancing treatment outcomes.

## 2 Methods

### 2.1 General information

A total of 96 patients with DS admitted to our spine department from September 2018 to December 2020 were retrospectively analyzed and divided into two groups based on the number of fixed segments: 64 patients with short-segment fixation (short-segment group) and 32 patients with long-segment fixation (long-segment group) (Figure S1).

The inclusion criteria were as follows: (1) patients with a diagnosis of DS confirmed by preoperative symptoms and imaging findings, treated with posterior short-segment decompression fusion internal fixation (fusion segment limited to the upper and lower ends of the laterally curved spine); (2) patients with a follow-up period of at least 1 year; and (3) patients with complete clinical and imaging data, including preoperative full-length frontal and lateral radiographs of the spine, postoperative lateral radiographs of the lumbar spine, and magnetic resonance imaging (MRI) preoperatively and at the final follow-up.

The exclusion criteria were as follows: (1) incomplete clinical information; (2) Parkinson’s disease, neuromuscular spondylitis, juvenile idiopathic spondylitis, or secondary congenital spondylitis; (3) complicated spinal infection, tuberculosis, trauma, or tumor; (4) history of previous spinal surgery; (5) inability to cooperate with follow-up or loss of follow-up information; (6) combined spinal fracture, spinal infection, spinal tumor, or ankylosing spondylitis; (7) history of previous lumbar spine surgery; and (8) previous anterior or lateral approach fusion of the lumbar spine.

### 2.2 Observation methods for preoperative and postoperative follow-up

Medical history data, clinical symptoms, signs, imaging data, and curative effect evaluation indices were collected for all patients preoperatively, as well as at 3, 6, and 12 months postoperatively and at the last follow-up (Table S1). To reduce errors and increase the reliability of the results, the average value from multiple measurements was used. All data were measured three times by the same physician, and the average value was taken.

### 2.3 Evaluation of efficacy

Surgical outcomes were assessed using visual analogue scores (VAS) to evaluate the severity of pre- and post-operative leg and low back pain in patients [11]. The Oswestry Dysfunction Index (ODI) was used to determine the severity of the pre- and post-operative spinal dysfunction of the patients. The ODI questionnaire consists of ten questions covering pain intensity, self-care, lifting, walking, sitting, sleep disturbances, sex life, social life, and travel, with six response options for each question. The minimum ODI score is calculated as follows: (score obtained/5 × number of questions answered) × 100%. A score of 0% indicates no functional impairment, while a score of 100% indicates complete functional impairment. The improvement rate of the ODI score is calculated using the formula: improvement rate = (1 – post-operative ODI score/pre-operative ODI score) × 100%. Improvement rates are classified as follows: an improvement of ≥75% is considered excellent, 50–75% is considered good, 25–50% is considered moderate, and <25% is considered poor [12].

### 2.4 Influencing factors related to osteoarthritis

ASD was observed in 10 of 64 patients with short-segment fixation, who were then divided into two groups: ASD (*n* = 10) and NASD (*n* = 54). The ASD group consisted of ten patients with ASD on the cranial side (15.6%), while the NASD group consisted of 54 patients without ASD on the caudal side (84.4%). This study included possible correlates, divided into three parts: (1) demographic factors: gender, age, smoking status (present or absent), body mass index (BMI), preoperative leg pain and low back pain VAS scores, and preoperative ODI scores; (2) surgical correlates: number of fused segments, operative segments, operative time, bleeding, and whether adjacent synapses were violated during surgery; and (3) imaging parameters: anterior lumbar lordosis (LL), sacral tilt angle (SS), pelvic tilt (PT) angle, PI angle, PI–LL, distance between the L1 lead line and S1 (LASD), coronal Cobb angle, and preoperative disc degeneration in adjacent segments (graded I–V according to the Pfirrmann et al. classification system). Patients classified as having grade ≥III were considered to have degenerative changes.

### 2.5 Measurement of imaging parameters

Cobb angle: measured in the coronal position as the angle between the tangent lines of the upper and lower endplates; LL: defined as the angle between the tangent line of the upper endplate of L1 and that of S1; SS: determined as the angle between the horizontal line and the tangent line of the upper endplate of S1; PT: calculated as the angle between the gravitational plumb line and the line connecting the midpoint of the upper endplate of S1 to the central midpoint of the femoral heads bilaterally; PI: measured as the angle between the line from the midpoint of the upper endplate of S1 to the central midpoint of the femoral heads bilaterally and the perpendicular line through the midpoint of the S1 upper endplate; PI–LL: represents the difference between the pelvic projection angle (PPA) and the lumbar anterior convexity angle, with a positive value taken; LASD: defined as the horizontal distance between the plumb line through the center of the L1 vertebra and the posterior superior edge of S1 (a positive value indicates an anterior position of the L1 plumb line, while a negative value indicates a posterior position). ASD imaging criteria: included anterior or posterior slip >3 mm; adjacent segmental disc height loss >3 mm; intervertebral angle >5° on flexion–extension lateral views; and the presence of spinal stenosis or disc herniation at adjacent levels on follow-up MRI.

All imaging data were measured three times by the same physician, and the mean value was used for analysis.

### 2.6 Assessment of lumbar disc degeneration

The MRI (T2W1) grading criteria for Pfirrmann disc degeneration are shown in Table S2.

### 2.7 Surgical techniques

Following general anesthesia with tracheal intubation, the patient is placed in the prone position, disinfected, and draped in a sterile fashion. A conventional lumbar midline dorsal incision is made, and the paraspinal muscles are stripped down to the outer surface of the articular eminence to expose the fixed segment. The vertebral lamina and articular eminence are also exposed. Using the apex of the “human” ridge as the reference point, an opening is made, a hole is drilled, the depth is measured, and the thread is tapped. A pedicle screw of the appropriate length and thickness is placed based on the measurements. The central canal and lateral saphenous fossa are decompressed within the fixed segment. The intervertebral discs and cartilage endplates are completely removed, and the space is fused with a trimmed autogenous bone block and an intervertebral fusion device. Depending on the preoperative spinal deformity, a curved metal rod is selected to support the concave side, and appropriate pressure is applied to the convex side to correct the scoliosis deformity. If necessary, the interbody fusion device is placed on the concave side of the vertebral apex. Fluoroscopy with a C-arm X-ray machine is used to confirm the satisfactory position of the internal fixation. The incision is then sutured, and a silicone ball drain is placed, which is removed 72 h postoperatively. Antibacterial medication is administered for 1–3 days to prevent infection. A lumbar brace was worn for 3 days postoperatively, and then immobilization with the brace continued for 3 months. After this period, the brace was removed, and the patient began appropriate functional exercise.

### 2.8 Statistical analysis

Data for all relevant variables in this study were analyzed using IBM SPSS Statistics for Windows (version 22.0; IBM Corp., Armonk, NY, USA). Continuous variables are expressed as the mean ± standard deviation. Continuous variables such as VAS score, ODI score, age, BMI, operative time, blood loss, LL, SS, PT, PI, LASD, PI–LL, and coronal Cobb angle were analyzed using the *t*-test. Categorical variables, including gender, smoking status, number of fused segments, operative segments, whether adjacent synapses were violated during surgery, and preoperative adjacent segment disc degeneration grade, were analyzed using the chi-square test. Significant differences between the two groups were determined by a *P*-value of <0.05, while highly significant differences were determined by a *P*-value of <0.05.


**Ethical approval:** The study was approved by the Ethics Committee of the General Hospital of Ningxia Medical University (No. 2017-131).

## 3 Results

### 3.1 Clinical data

There was no significant difference in age, height, weight, or BMI between the two groups (*P* > 0.05), as shown in Table 1.

**Table 1:** Comparison of preoperative basic conditions between the two groups

	Short-segment group	Long-segment group	*t*	*P*
Age	63.31 ± 7.49	64.00 ± 5.97	0.051	0.824
Height	162.19 ± 11.42	165.00 ± 8.17	0.383	0.543
Weight	68.00 ± 15.23	68.75 ± 10.71	0.015	0.902
BMI	25.78 ± 5.06	25.37 ± 4.64	0.036	0.851
Apical rotation Nash Moe	1.88 ± 0.50	2.00 ± 0.53	7.971	0.095
Cobb angle (°)	11.08 ± 5.17	11.87 ± 6.17	1.449	0.241
Coronal imbalance value (mm)	14.39 ± 3.66	16.95 ± 7.17	1.364	0.255
Lateral sliding of apical vertebra (mm)	6.54 ± 4.94	9.24 ± 7.36	1.148	0.296
Lumbar lordosis (°)	18.13 ± 12.40	23.76 ± 11.37	1.160	0.293
Sagittal imbalance value (mm)	33.83 ± 17.90	27.47 ± 13.87	0.771	0.390
Pelvic projection angle (°)	56.33 ± 9.70	62.18 ± 10.00	1.901	0.182
Pelvic inclination angle (°)	44.37 ± 7.22	42.97 ± 6.42	0.215	0.648
Sacral inclination angle (°)	42.66 ± 11.98	44.31 ± 9.41	0.115	0.738
Proximal intervertebral space height *A* (mm)	10.62 ± 2.25	9.09 ± 2.04	2.619	0.120
Proximal intervertebral space height *B* (mm)	7.28 ± 2.20	6.96 ± 1.27	0.147	0.705
Posterior diameter of proximal vertebral precursor *D* (mm)	41.27 ± 5.29	40.24 ± 4.20	0.231	0.636
Proximal ratio (*A* + *B*)/2/*D*	0.22 ± 0.06	0.33 ± 0.37	1.425	0.245

Several parameters were not statistically different between the two groups of patients preoperatively. These included apical rotation Nash Moe, Cobb angle, coronal imbalance value, lateral sliding of the apical vertebra, LL, sagittal imbalance value, PPA, pelvic inclination (PI) angle, sacral inclination angle, proximal intervertebral space heights *A* and *B*, posterior diameter of the proximal vertebral precursor *D*, and proximal ratio (*P* > 0.05). The details are shown in Table 1.

### 3.2 Data measurement for both groups of patients

The two groups were followed up for 6 months, 1 year, and 2 years postoperatively. There was a significant difference between the two groups in bleeding volume, operation time, length of stay, apical rotation Nash Moe, Cobb angle, coronal imbalance value, lateral sliding of the apical vertebra, sacral inclination angle, and Cobb angle of the upper and fixed vertebrae (*P* < 0.05), as shown in Table 2.

**Table 2:** Comparison of follow-up results of the two groups at 6 months, 1 year, and 2 years after operation

	Short-segment group	Long-segment group	*t*	*P*
**After operation**				
Bleeding volume (mL)	293.75 ± 104.68	878.75 ± 711.87	3.289	<0.05
Operation time (min)	190.31 ± 53.87	264.38 ± 108.15	2.266	<0.05
Length of stay (day)	26.38 ± 8.49	30.63 ± 10.13	2.583	<0.05
Apical rotation Nash Moe	1.50 ± 0.52	1.75 ± 0.89	3.321	<0.05
Cobb angle (°)	8.12 ± 3.23	9.79 ± 4.35	6.038	<0.05
Coronal imbalance value (mm)	49.64 ± 8.98	47.67 ± 15.58	2.629	<0.05
Lateral sliding of apical vertebra (mm)	5.48 ± 2.59	7.49 ± 5.47	3.654	<0.05
Lumbar lordosis (°)	50.77 ± 12.35	50.86 ± 15.15	0.015	0.987
Sagittal imbalance value (mm)	53.31 ± 7.18	52.38 ± 7.15	6.519	<0.05
Pelvic projection angle (°)	46.46 ± 10.19	52.10 ± 8.72	5.128	<0.05
Pelvic inclination angle (°)	24.07 ± 11.96	24.89 ± 7.4	4.108	<0.05
Sacral inclination angle (°)	26.7 ± 10.13	25.97 ± 11.24	2.983	<0.05
Cobb angle of upper fixed vertebra (°)	4.94 ± 2.75	3.84 ± 4.62	14.77	<0.05
Cobb angle of lower fixed vertebra (°)	5.12 ± 2.91	3.92 ± 3.52	11.75	<0.05
**Six months after operation**				
Apical rotation Nash Moe	1.94 ± 0.77	3.01 ± 0.93	2.998	<0.05
Cobb angle (°)	7.01 ± 4.34	7.66 ± 2.94	6.386	<0.05
Coronal imbalance value (mm)	11.27 ± 6.12	20.54 ± 8.23	3.120	<0.05
Lateral sliding of apical vertebra (mm)	9.28 ± 3.70	13.52 ± 2.64	2.881	<0.05
Lumbar lordosis (°)	31.15 ± 11.56	31.12 ± 12.21	0.005	0.995
Sagittal imbalance value (mm)	15.24 ± 2.94	35.21 ± 3.04	15.52	<0.05
Pelvic projection angle (°)	25.43 ± 9.17	40.21 ± 9.54	3.674	<0.05
Pelvic inclination angle (°)	20.54 ± 1.26	36.21 ± 10.95	5.778	<0.05
Sacral inclination angle (°)	19.25 ± 6.90	33.64 ± 8.47	4.469	<0.05
Proximal intervertebral space height *A* (mm)	7.56 ± 3.16	8.40 ± 4.20	0.550	0.587
Proximal intervertebral space height *B* (mm)	4.08 ± 2.26	5.15 ± 2.76	1.017	0.320
Posterior diameter of proximal vertebral precursor *D* (mm)	28.99 ± 12.12	20.53 ± 15.85	1.456	0.159
Proximal ratio (*A* + *B*)/2/*D*	0.44 ± 0.85	0.48 ± 0.39	0.125	0.901
Distal intervertebral space height *A* (mm)	9.69 ± 3.47	9.03 ± 2.16	0.489	0.629
Distal intervertebral space height *B* (mm)	8.51 ± 2.62	7.91 ± 4.82	0.398	0.693
Posterior diameter of distal vertebral precursor *D* (mm)	29.75 ± 10.69	25.43 ± 13.98	0.842	0.408
Distal ratio (*A* + *B*)/2/*D*	0.35 ± 0.17	0.45 ± 0.24	1.184	0.248
Cobb angle of upper fixed vertebra (°)	20.13 ± 9.44	38.23 ± 11.16	5.785	<0.05
Cobb angle of lower fixed vertebra (°)	18.21 ± 9.92	35.72 ± 6.69	4.484	<0.05
JOA low back pain score	13.13 ± 2.83	25.12 ± 2.62	18.37	<0.05
ODI score of low back pain	35.70 ± 18.07	41.10 ± 19.65	3.156	<0.05
Lumbar spine stiffness disability index score	2.01 ± 0.41	2.59 ± 0.46	3.140	<0.05
**One year after operation**				
Apical rotation Nash Moe	1.12 ± 0.70	2.38 ± 0.74	4.081	<0.05
Cobb angle (°)	5.52 ± 3.26	6.25 ± 3.56	7.207	<0.05
Coronal imbalance value (mm)	8.61 ± 7.8	9.36 ± 7.92	3.167	<0.05
Lateral sliding of apical vertebra (mm)	6.27 ± 1.84	9.17 ± 2.16	3.439	<0.05
Lumbar lordosis (°)	49.41 ± 10.89	47.94 ± 16.75	0.260	0.797
Sagittal imbalance value (mm)	11.47 ± 9.25	18.76 ± 8.98	4.357	<0.05
Pelvic projection angle (°)	15.54 ± 6.23	25.76 ± 6.25	7.488	<0.05
Pelvic inclination angle (°)	16.48 ± 6.32	22.75 ± 13.3	4.112	<0.05
Sacral inclination angle (°)	15.32 ± 4.54	25.67 ± 5.01	5.091	<0.05
Proximal intervertebral space height *A* (mm)	16.68 ± 5.85	16.46 ± 6.01	0.086	0.932
Proximal intervertebral space height *B* (mm)	17.56 ± 7.64	17.99 ± 9.53	0.119	0.905
Posterior diameter of proximal vertebral precursor *D* (mm)	25.19 ± 10.55	31.67 ± 12.46	1.337	0.194
Proximal ratio (*A* + *B*)/2/*D*	0.81 ± 0.47	0.73 ± 0.63	0.351	0.728
Distal intervertebral space height *A* (mm)	12.16 ± 3.39	10.26 ± 4.04	1.216	0.237
Distal intervertebral space height *B* (mm)	12.41 ± 5.15	14.09 ± 5.87	0.719	0.479
Posterior diameter of distal vertebral precursor *D* (mm)	29.54 ± 12.61	25.86 ± 14.51	0.641	0.527
Distal ratio (*A* + *B*)/2/*D*	0.5 ± 0.27	0.63 ± 0.39	0.958	0.348
Cobb angle of upper fixed vertebra (°)	16.75 ± 7.69	26.24 ± 11.29	5.005	<0.05
Cobb angle of lower fixed vertebra (°)	15.35 ± 6.38	23.72 ± 9.77	5.565	<0.05
JOA low back pain score	22.32 ± 2.21	26.78 ± 2.6	4.400	<0.05
ODI score of low back pain	32.5 ± 9.44	45.12 ± 6.74	6.023	<0.05
Lumbar spine stiffness disability index score	1.56 ± 0.51	2.39 ± 0.52	3.735	<0.05
**Two years after operation**				
Apical rotation Nash Moe	0.75 ± 0.58	1.96 ± 0.52	4.976	<0.05
Cobb angle (°)	4.37 ± 3.40	5.42 ± 4.67	6.257	<0.05
Coronal imbalance value (mm)	4.32 ± 3.73	15.53 ± 2.82	7.468	<0.05
Lateral sliding of apical vertebra (mm)	5.06 ± 0.88	7.89 ± 0.96	6.748	<0.05
Lumbar lordosis (°)	40.28 ± 11.56	42.34 ± 11.94	0.407	0.687
Sagittal imbalance value (mm)	10.23 ± 5.85	19.52 ± 5.93	3.651	<0.05
Pelvic projection angle (°)	18.52 ± 2.71	21.36 ± 3.46	5.320	<0.05
Pelvic inclination angle (°)	15.32 ± 8.39	22.75 ± 7.19	3.864	<0.05
Sacral inclination angle (°)	10.23 ± 4.34	18.24 ± 4.71	4.147	<0.05
Proximal intervertebral space height *A* (mm)	14.60 ± 7.97	14.31 ± 7.37	0.086	0.932
Proximal intervertebral space height *B* (mm)	17.84 ± 6.89	15.23 ± 7.67	0.843	0.408
Posterior diameter of proximal vertebral precursor *D* (mm)	22.04 ± 10.91	29.57 ± 12.38	1.526	0.141
Proximal ratio (*A* + *B*)/2/*D*	0.93 ± 0.57	0.67 ± 0.47	1.112	0.278
Distal intervertebral space height *A* (mm)	12.51 ± 4.41	11.37 ± 4.12	0.609	0.548
Distal intervertebral space height *B* (mm)	12.70 ± 5.63	10.25 ± 6.18	0.973	0.340
Posterior diameter of distal vertebral precursor *D* (mm)	30.74 ± 9.15	30.63 ± 11.15	0.025	0.979
Distal ratio (*A* + *B*)/2/*D*	0.43 ± 0.14	0.40 ± 0.18	0.450	0.656
Cobb angle of upper fixed vertebra (°)	15.26 ± 6.30	12.75 ± 6.39	5.287	<0.05
Cobb angle of lower fixed vertebra (°)	14.67 ± 5.21	9.31 ± 10.64	4.579	<0.05
JOA low back pain score	18.21 ± 2.42	5.73 ± 2.51	7.091	<0.05
ODI score of low back pain	7.65 ± 3.51	6.13 ± 4.16	5.252	<0.05
Lumbar spine stiffness disability index score	1.50 ± 0.52	3.65 ± 0.74	8.291	<0.05

Six months postoperatively, there was a significant difference between the two groups in apical rotation Nash Moe, Cobb angle, coronal imbalance value, lateral sliding of the apical vertebra, sacral inclination angle, Cobb angle of the upper and lower fixed vertebrae, Japanese Orthopaedic Association (JOA) and ODI low back pain scores, and lumbar spine stiffness disability index score (*P* < 0.05), as shown in Table 2.

One year postoperatively, there was a significant difference between the two groups in apical rotation Nash Moe, Cobb angle, coronal imbalance value, lateral sliding of the apical vertebra, sacral inclination angle, Cobb angle of the upper and lower fixed vertebrae, JOA and ODI low back pain scores, and lumbar spine stiffness disability index score (*P* < 0.05), as shown in Table 2.

Two years postoperatively, there was a significant difference between the two groups in apical rotation Nash Moe, Cobb angle, coronal imbalance value, lateral sliding of the apical vertebra, sacral inclination angle, Cobb angle of the upper and lower fixed vertebrae, JOA and ODI low back pain scores, and lumbar spine stiffness disability index score (*P* < 0.05), as shown in Table 2.

A comparison of treatment effects in patients in the short-segment group preoperatively and 6 months postoperatively revealed significant differences in coronal imbalance value, LL, lateral sliding of the apical vertebra, sacral inclination angle, proximal intervertebral space heights *A* and *B*, posterior diameter of the proximal and distal vertebral precursors *D*, distal ratio, Cobb angle of the upper fixed vertebra, JOA and ODI low back pain scores, and lumbar spine stiffness disability index score (*P* < 0.05), as shown in Table 3.

**Table 3:** Comparison of patients in short-segment group before operation and 6 months after operation

	Preoperative	Six months after operation	*t*	*P*
Apical rotation Nash Moe	1.60 ± 0.50	1.94 ± 0.77	1.481	0.148
Cobb angle (°)	8.80 ± 5.17	9.23 ± 4.34	0.254	0.800
Coronal imbalance value (mm)	55.30 ± 3.66	11.27 ± 6.12	24.700	<0.05
Lateral sliding of apical vertebra (mm)	6.54 ± 4.94	9.28 ± 3.70	0.829	0.413
Lumbar lordosis (°)	18.13 ± 12.40	31.15 ± 11.56	2.678	<0.05
Sagittal imbalance value (mm)	33.83 ± 17.90	15.24 ± 2.94	4.099	<0.05
Pelvic projection angle (°)	56.33 ± 9.70	25.43 ± 9.17	9.260	<0.05
Pelvic inclination angle (°)	44.37 ± 7.22	20.54 ± 1.26	13.010	<0.05
Sacral inclination angle (°)	42.66 ± 11.98	19.25 ± 6.90	6.773	<0.05
Proximal intervertebral space height *A* (mm)	10.62 ± 2.25	7.56 ± 3.16	3.155	<0.05
Proximal intervertebral space height *B* (mm)	7.28 ± 2.20	4.08 ± 2.26	4.058	<0.05
Posterior diameter of proximal vertebral precursor *D* (mm)	41.27 ± 5.29	28.99 ± 12.12	3.714	<0.05
Proximal ratio (*A* + *B*)/2/*D*	0.22 ± 0.06	0.44 ± 0.85	1.033	0.309
Distal intervertebral space height *A* (mm)	10.23 ± 2.84	9.69 ± 3.47	0.481	0.633
Distal intervertebral space height *B* (mm)	7.37 ± 2.29	8.51 ± 2.62	1.310	0.199
Posterior diameter of distal vertebral precursor *D* (mm)	42.76 ± 5.76	29.75 ± 10.69	4.286	<0.05
Distal ratio (*A* + *B*)/2/*D*	0.21 ± 0.04	0.35 ± 0.17	3.207	<0.05
Cobb angle of upper fixed vertebra (°)	4.95 ± 3.33	20.13 ± 9.44	4.338	<0.05
Cobb angle of lower fixed vertebra (°)	5.20 ± 2.90	18.21 ± 9.92	1.293	0.205
JOA low back pain score	6.31 ± 2.36	13.13 ± 2.83	13.110	<0.05
ODI score of low back pain	69.44 ± 8.94	35.70 ± 18.07	6.694	<0.05
Lumbar spine stiffness disability index score	2.50 ± 0.52	2.01 ± 0.41	2.960	<0.05

A comparison of treatment effects in patients in the short-segment group preoperatively and 1 year postoperatively revealed significant differences in apical rotation Nash Moe, coronal imbalance value, lateral sliding of the apical vertebra, sagittal imbalance value, PPA, PI angle, sacral inclination angle, proximal intervertebral space heights *A* and *B*, posterior diameter of the proximal and distal vertebral precursors *D*, proximal and distal ratios, distal intervertebral space heights *A* and *B*, Cobb angle of the upper and lower fixed vertebrae, JOA and ODI low back pain scores, and lumbar spine stiffness disability index score (*P* < 0.05), as shown in Table 4.

**Table 4:** Comparison of patients in short-segment group before operation and 1 year after operation

	Preoperative	One year after operation	*t*	*P*
Apical rotation Nash Moe	1.60 ± 0.50	1.12 ± 0.70	2.232	<0.05
Cobb angle (°)	8.8 ± 5.17	7.79 ± 3.26	0.661	0.513
Coronal imbalance value (mm)	55.30 ± 3.66	8.61 ± 7.80	21.680	<0.05
Lateral sliding of apical vertebra (mm)	6.54 ± 4.94	6.27 ± 1.84	3.255	<0.05
Lumbar lordosis (°)	18.13 ± 12.4	49.41 ± 10.89	1.675	0.104
Sagittal imbalance value (mm)	33.83 ± 17.9	11.47 ± 9.25	4.439	<0.05
Pelvic projection angle (°)	56.33 ± 9.7	15.54 ± 6.23	14.15	<0.05
Pelvic inclination angle (°)	44.37 ± 7.22	16.48 ± 6.32	11.63	<0.05
Sacral inclination angle (°)	42.66 ± 11.98	15.32 ± 4.54	8.536	<0.05
Proximal intervertebral space height *A* (mm)	10.62 ± 2.25	16.68 ± 5.85	3.867	<0.05
Proximal intervertebral space height *B* (mm)	7.28 ± 2.20	17.56 ± 7.64	5.172	<0.05
Posterior diameter of proximal vertebral precursor *D* (mm)	41.27 ± 5.29	25.19 ± 10.55	5.450	<0.05
Proximal ratio (*A* + *B*)/2/*D*	0.22 ± 0.06	0.81 ± 0.47	4.981	<0.05
Distal intervertebral space height *A* (mm)	10.23 ± 2.84	12.16 ± 3.39	1.746	<0.05
Distal intervertebral space height *B* (mm)	7.37 ± 2.29	12.41 ± 5.15	3.577	<0.05
Posterior diameter of distal vertebral precursor *D* (mm)	42.76 ± 5.76	29.54 ± 12.61	3.814	<0.05
Distal ratio (*A* + *B*)/2/*D*	0.21 ± 0.04	0.50 ± 0.27	4.250	<0.05
Cobb angle of upper fixed vertebra (°)	4.95 ± 3.33	16.75 ± 7.69	6.406	<0.05
Cobb angle of lower fixed vertebra (°)	5.20 ± 2.90	15.35 ± 6.38	3.539	<0.05
JOA low back pain score	6.31 ± 2.36	22.32 ± 2.21	3.575	<0.05
ODI score of low back pain	69.44 ± 8.94	32.5 ± 9.44	11.360	<0.05
Lumbar spine stiffness disability index score	2.5 ± 0.52	1.56 ± 0.51	5.162	<0.05

A comparison of treatment effects in patients in the short-segment group preoperatively and 2 years postoperatively revealed significant differences in apical rotation Nash Moe, Cobb angle, coronal imbalance value, lateral sliding of the apical vertebra, sagittal imbalance value, PPA, PI angle, sacral inclination angle, proximal intervertebral space heights *A* and *B*, posterior diameter of the proximal and distal vertebral precursors *D*, proximal and distal ratios, distal intervertebral space heights *A* and *B*, Cobb angle of the upper and lower fixed vertebrae, JOA and ODI low back pain scores, and lumbar spine stiffness disability index score (*P* < 0.05), as shown in Table 5.

**Table 5:** Comparison of patients in short-segment group before operation and 2 years after operation

	Preoperative	Two years after operation	*t*	*P*
Apical rotation Nash Moe	1.60 ± 0.50	0.75 ± 0.58	4.440	<0.05
Cobb angle (°)	8.80 ± 5.17	5.33 ± 3.40	2.243	<0.05
Coronal imbalance value (mm)	55.30 ± 3.66	4.32 ± 3.73	39.020	<0.05
Lateral sliding of apical vertebra (mm)	6.54 ± 4.94	5.06 ± 0.88	4.101	<0.05
Lumbar lordosis (°)	18.13 ± 12.40	40.28 ± 11.56	0.5238	0.604
Sagittal imbalance value (mm)	33.83 ± 17.90	10.23 ± 5.85	5.013	<0.05
Pelvic projection angle (°)	56.33 ± 9.7	18.52 ± 2.71	15.020	<0.05
Pelvic inclination angle (°)	44.37 ± 7.22	15.32 ± 8.39	10.500	<0.05
Sacral inclination angle (°)	42.66 ± 11.98	10.23 ± 4.34	10.180	<0.05
Proximal intervertebral space height *A* (mm)	10.62 ± 2.25	14.6 ± 7.97	1.922	0.064
Proximal intervertebral space height *B* (mm)	7.28 ± 2.20	17.84 ± 6.89	5.840	<0.05
Posterior diameter of proximal vertebral precursor *D* (mm)	41.27 ± 5.29	22.04 ± 10.91	6.344	<0.05
Proximal ratio (*A* + *B*)/2/*D*	0.22 ± 0.06	0.93 ± 0.57	4.955	<0.05
Distal intervertebral space height *A* (mm)	10.23 ± 2.84	12.51 ± 4.41	1.739	0.092
Distal intervertebral space height *B* (mm)	7.37 ± 2.29	12.70 ± 5.63	3.508	<0.05
Posterior diameter of distal vertebral precursor *D* (mm)	42.76 ± 5.76	30.74 ± 9.15	4.447	<0.05
Distal ratio (*A* + *B*)/2/*D*	0.21 ± 0.04	0.43 ± 0.14	6.044	<0.05
Cobb angle of upper fixed vertebra (°)	4.95 ± 3.33	15.26 ± 6.30	7.916	<0.05
Cobb angle of lower fixed vertebra (°)	5.20 ± 2.90	14.67 ± 5.21	4.615	<0.05
JOA low back pain score	6.31 ± 2.36	18.21 ± 2.42	8.283	<0.05
ODI score of low back pain	69.44 ± 8.94	7.65 ± 3.51	25.730	<0.05
Lumbar spine stiffness disability index score	2.50 ± 0.52	1.50 ± 0.52	5.439	<0.05

### 3.3 Factors associated with osteoarthritis

In the above study, we found that short-segment fixation was more effective in DS. To further explore the factors associated with ASD, we examined ASD in 10 out of 64 patients with short-segment fixation. These patients were divided into the ASD (*n* = 10) and NASD (*n* = 54) groups. In the ASD group, 10 patients (15.6%) had ASD on the cranial side. In the NASD group, 54 patients (84.4%) had no ASD on the caudal side. When comparing the ASD and NASD groups, the differences in demographic factors such as mean follow-up time, gender, age, smoking status, BMI, preoperative low back pain VAS score, preoperative leg pain VAS score, and preoperative ODI score were not statistically significant (*P* > 0.05), as shown in Table 6.

**Table 6:** Comparison of demographic parameters of patients in the ASD and N-ASD groups

Related factors	ASD group (*n* = 10)	NASD group (*n* = 54)	*P*
Duration of follow-up visits (months)	27.5 ± 6.4	27.3 ± 11.8	0.985
Age (years)	66.8 ± 3.9	64.3 ± 7.6	0.467
Gender			0.146
Male	2	20	
Female	8	34	
Smoking			0.517
Yes	3	7	
No	7	47	
BMI	25.5 ± 3.4	24.1 ± 2.8	0.302
Pre-operative low back pain VAS	6.0 ± 0.5	5.3 ± 1.1	0.395
Pre-operative leg pain VAS	4.2 ± 1.4	3.9 ± 1.3	0.687
Pre-operative ODI (%)	53.7 ± 3.5	51.5 ± 4.6	0.321

Statistical analysis of surgically relevant variables showed no statistically significant differences between the two groups in the number of fused segments, operative segments, operative time, bleeding, and other surgical variables. No statistical analysis was carried out on adjacent synovial joints, as none occurred intraoperatively in either group (Table 7).

**Table 7:** Comparison of surgery-related factors between patients in the ASD and NASD groups

Related factors	ASD group (*n* = 10)	NASD group (*n* = 54)	*P*
Number of fusions			0.063
1	0	24	
2	8	21	
3	2	9	
Fusion of segments			0.450
L3–L4	0	4	
L4–L5	0	15	
L5–S1	0	3	
L2–L4	0	5	
L3–L5	6	8	
L4–S1	3	7	
L1–L4	0	2	
L2–L5	1	6	
L3–S1	0	4	
Operating time (min)	218.1 ± 16.8	196.5 ± 52.1	0.334
Bleeding volume (mL)	829.6 ± 134.3	756.1 ± 374.8	0.639

When comparing imaging factors between the two groups, preoperative PT (33.4 ± 9.5 vs 20.8 ± 8.1; *P* = 0.003), preoperative PI (54.8 ± 8.0 vs 45.6 ± 6.7; *P* = 0.006), preoperative PI–LL (32.2 ± 15.6 vs 17.9 ± 12.2; *P* = 0.0018), and preoperative coronal Cobb angle (28.4 ± 4.1 vs 19.6 ± 6.5; *P* = 0.009) were higher in patients with adjacent vertebral involvement compared to those without (*P* < 0.05), as shown in Table 8.

**Table 8:** Comparison of imaging parameters between patients in the ASD and NASD groups

Related factors	ASD group (*n* = 10)	NASD group (*n* = 54)	*P*
LL (°)	22.6 ± 17.0	29.4 ± 17.8	0.405
PI (°)	54.8 ± 8.0	45.6 ± 6.7	0.006*
PT (°)	33.4 ± 9.5	20.8 ± 8.1	0.003*
SS (°)	21.6 ± 10.5	24.7 ± 9.4	0.503
PI–LL (°)	32.2 ± 15.6	17.9 ± 12.2	0.018*
LASD (mm)	13.3 ± 17.4	14.8 ± 25.3	0.866
Cobb (°)	28.4 ± 4.1	19.6 ± 6.5	0.009*
Degeneration of adjacent intervertebral discs			0.524
≥Class III			0.524
Yes	8	49	
No	2	5	

Note: *P < 0.05 and **P < 0.01 compared to the ASD group; lordosis; SS, sacral tilt angle; PT, pelvic tilt angle; PI, pelvic projection angle; PI–LL, degree of matching of pelvic projection angle to lumbar lordosis; LASD, distance between L1 lead line and S1.

## 4 Discussion

ADS is primarily a spinal deformity seen in middle-aged and elderly patients without a history of scoliosis. Degenerative changes, such as those affecting the lumbar disc and facet joints, can lead to scoliosis. This condition mostly affects the thoracolumbar and lumbar segments, primarily due to asymmetric degeneration of the lumbar spine [13].

Asymmetric degeneration of the intervertebral disc and facet joint leads to an asymmetric load on the spine segment, resulting in asymmetric deformity. This, in turn, exacerbates the original asymmetric degeneration and load, creating a vicious cycle that ultimately leads to DS [14,15]. In this condition, low back pain and neurogenic claudication are the main symptoms. The pain, primarily low back and lower limb pain, is due to muscle spasticity caused by scoliosis deformity and radiating pain from nerve root compression [16]. Neurogenic pain is mainly caused by the compression of both convex and concave nerve roots due to intervertebral disc protrusion, lateral recess stenosis, and articular process hyperplasia in DS [17]. Patients with DS often present with low back pain, leg pain, and spinal imbalance. Lameness or root pain is caused by spinal stenosis or rotational subluxation, where nerve roots are compressed due to the blending of the concave pedicle or stretching of the convex pedicle. Correcting rotational subluxation can help decompress nerve roots and reduce leg pain [18]. Sagittal plane imbalance leads to muscle strength imbalance and low back pain [19]. Therefore, it is recommended to restore the sagittal plane imbalance. The primary goal of surgical treatment for DS is to relieve symptoms, including leg and back pain, with deformity correction as a secondary objective [20].

Surgical treatments for ADS include simple decompression, post-decompression long- and short-segment fusion, and fixation. To avoid spinal instability caused by laminectomy, internal fixation and fusion surgery are often combined with decompression to maintain spinal stability and overall balance after reconstruction [21,22]. Patients with ADS often have osteoporosis, which increases surgical complications and difficulty. Therefore, the choice between long-segment and short-segment fixation for fusion remains highly controversial as surgeons strive to ensure therapeutic effectiveness while minimizing injuries [23].

At the last follow-up, the ODI score for the short-segment group was 7.65 ± 3.51, while for the long-segment group, it was 16.13 ± 4.16, indicating a significant difference between the two groups. Although both groups showed improvement, the degree of improvement significantly differed between them. Short-segment and long-segment surgeries have distinct clinical advantages. Short-segment surgery entails less trauma and shorter recovery times, whereas long-segment surgery imposes greater trauma on the tissues surrounding the injured site, potentially impeding nerve and muscle recovery and prolonging healing time.

If there is no obvious imbalance in the coronal and sagittal planes of the patient, fixation may only be required within the lateral bending range. Short-segment fixation and fusion can be utilized to address the responsible decompression segment while stabilizing the spine, thereby preventing scoliosis progression and iatrogenic spine instability-induced decompensation. Considering the findings of this study, if there are multiple underlying medical conditions preoperatively and imaging indicates a minimal Cobb angle (<20°) or no significant imbalance between the coronal and sagittal spinal positions, responsible segmental decompression with short-segmental fusion and internal fixation can be considered to alleviate symptoms. Treatment in such cases may not necessarily entail excessive pursuit of deformity correction [21].

To achieve optimal surgical outcomes, restoring sagittal plane balance is crucially important. The restoration of lumbar physiological lordosis significantly impacts the efficacy of surgery. Additionally, the fatigue and spasms of paraspinal muscles resulting from coronal plane imbalance can induce axial pain in patients, and a Cobb angle ≥30° is also a risk factor for DS progression [22]. In line with the findings of this study, in cases of severe spinal deformity characterized by a Cobb angle >20°, accompanied by significant loss of LL and sacral inclination angle, as well as three-dimensional spinal deformity, long-segment internal fixation and fusion may be preferred. This approach aims to achieve superior deformity correction, maintain spine stability, and yield satisfactory surgical outcomes.

Overall, the approach to treating patients with DS depends on the severity of the condition. For those with a small coronal Cobb angle, short-segment decompressive-fusion internal fixation is typically recommended. Conversely, patients with large coronal Cobb angles and significant sagittal and coronal imbalances usually require long-segment decompressive fusion internal fixation. However, it is crucial to consider that DS commonly occurs alongside multiple medical comorbidities in older patients, which can increase the risk of perioperative complications. Therefore, extending the fusion length may further elevate these risks. Presently, posterior short-segment lumbar fixation and fusion represent a critical surgical approach for managing DS [7]. In a meta-analysis comparing short-segment fusion to long-segment fusion for balanced DS, Lee et al. [24] found comparable decreases in patient ODI scores after 2 years of follow-up for both procedures. Notably, the short-fusion group exhibited reduced bleeding and shorter operative time compared to the long-fusion group. Patients undergoing short-segment lumbar fixation and fusion in this study demonstrated varying degrees of improvement in VAS and ODI scores for low back pain at 3 months postoperatively and during the final follow-up compared to the preoperative measures. Despite the favorable clinical outcomes associated with posterior short-segment lumbar fixation and fusion, it is important to acknowledge the potential complications, including implant failure, pseudoarthrosis, sagittal spine imbalance, and ASD. Additionally, the procedure alters the normal biomechanics of the spine by rigidly fusing segments, thereby increasing mechanical stress on adjacent segments and accelerating the degeneration of adjacent discs. Consequently, the development of ASD postoperatively significantly influences the need for reoperation after spinal fusion.

This retrospective study aimed to assess the incidence of ASD and its possible correlates in patients with DS who underwent lumbar fixed fusion. A total of 96 patients were included in the analysis, among whom 15 (15.6%) developed ASD. Notably, imaging degeneration of the adjacent segmental disc was frequently observed, although it did not always manifest with accompanying clinical symptoms. Park et al. reported that over a minimum follow-up period of 5 years, 42.6% of patients exhibited imaging evidence of ASD, with 30.3% experiencing symptomatic ASD [25]. Prior investigations have documented varying rates of ASD following lumbar fusion, ranging from 5 to 27%. In this study, ASD was defined as symptomatic ASD, with the study population exclusively comprising adult patients with DS. The observed incidence of symptomatic ASD in our study, at 15.6%, aligns closely with previously reported rates.

Debate persists regarding the influence of pre-existing disc degeneration on the onset of ASD. Studies have suggested a correlation between preoperative disc degeneration and the subsequent development of symptomatic ASD postoperatively. Clinical case studies and biomechanical analyses have consistently shown that postoperative ASD tends to be more prevalent in patients with advanced preoperative disc degeneration [26–28]. However, a minority of authors have suggested that postoperative symptomatic ASD may not be associated with pre-existing disc degeneration [29]. In our study, we found no association between pre-existing disc degeneration and the occurrence of postoperative symptomatic ASD. This outcome might be attributed to the presence of DS in our study cohort. The prevalence of preoperative disc degeneration in the adjacent segments did not significantly differ between the two groups in this study. Fixed fusion procedures lead to diminished elasticity and increased stiffness in the lumbar segment, resulting in biomechanical alterations in adjacent motion segments. These changes include stress concentration, increased segmental mobility, and elevated intradiscal pressure.

Following lumbar fusion surgery, biomechanical changes can induce progressive degeneration in healthy, mobile segments adjacent to the fusion site. Additionally, discs that have already degenerated in adjacent fused segments are predisposed to further degeneration postoperatively due to inherent functional decline and biomechanical changes. This progression can result in symptomatic ASD [30]. Consequently, the selection of the superior fusion segment becomes contentious, particularly in cases where there is significant disc degeneration in the adjacent segment compared to the targeted fused segment. Surgeons should consider preoperative disc degeneration in the adjacent fused segment when devising the surgical fusion plan to optimize patient outcomes.

In recent years, the impact of sagittal imbalance on the occurrence of ASD has garnered significant scholarly interest. Studies have consistently demonstrated an association between poor sagittal alignment and the development of ASD. Senteler et al. [31] reported that individuals with a high PI and reduced LL were predisposed to ASD, with those exhibiting a PI–LL mismatch (≥10°) having a tenfold higher risk of ASD compared to those without such a mismatch. Nakashima et al. similarly concluded that a high PI was significantly associated with the development of ASD. Phan et al. [32] found that preoperative PT and PI–LL were larger, while SS and LL were smaller in patients with postoperative ASD compared to controls, indicating significant correlations between changes in sagittal parameters (PT, SS, PI–LL, and LL) and the development of ASD. Moreover, Saitoh et al. found that preoperative LL was significantly lower and PT was significantly higher in the postoperative ASD group compared to controls. Additionally, they noted that 75% of patients with ASD exhibited a PI–LL mismatch (≥10°), highlighting high preoperative PT, low LL, and a PI–LL mismatch as significant risk factors for the development of postoperative ASD [33].

In this study, we observed no statistical difference in preoperative SS and LL values between the ASD and NASD groups. However, significant differences were noted in preoperative PI and PT between the ASD and NASD groups. Additionally, the preoperative PI–LL values exhibited a significant difference between the two groups, with patients in the ASD group demonstrating significantly higher preoperative PI, PT, and PI–LL values compared to the NASD group. These findings suggest a potential association between elevated preoperative PI, PT, and PI–LL values and the onset of ASD. Our study results are consistent with those reported by Phan and Matsumoto et al., underscoring the importance of further investigations with larger sample sizes to elucidate the relationship between spinal-pelvic imaging parameters and ASD. Bagheri et al. concluded that the distance between the L1 lead line and the posterior superior angle of S1 (LASD) was predictive of the development of ASD after lumbar fusion [34]. Lee and Park showed that preoperatively, patients with negative LASD were 5.6 times more likely to develop ASD postoperatively than those with positive LASD. However, LASD values did not exhibit statistical significance between the ASD and NASD groups in our analysis [12]. Few studies have explored the correlation between preoperative coronal Cobb angle and the occurrence of ASD following short-segment decompression fusion internal fixation, commonly employed in patients with minor Cobb angles or mild lateral displacement. Kim et al. found that long-segment internal fusion was more effective in correcting scoliosis angles and coronal imbalances, particularly in patients with Cobb angles >25° [35]. In our study, patients in the ASD group exhibited a significantly larger preoperative coronal Cobb angle compared to the NASD group, suggesting that a substantial preoperative coronal Cobb angle may contribute to the development of ASD following short-segment decompression fusion internal fixation in patients with DS.

Our study has several limitations. First, it was retrospective in nature, precluding the collection of prospective data and potentially introducing selective bias due to the loss of imaging data. Second, the sample size was insufficient to facilitate accurate regression analysis of the variables of interest and their relationship to ASD. Therefore, expanding the sample size is essential for further studies to delve deeper into this association. Third, the mean follow-up period in our study was relatively short, providing only interim efficacy data. Future investigations with longer follow-up periods are necessary to establish definitive conclusions regarding the incidence of ASD and its associated factors. Lastly, the findings of our study are of limited scope and applicability, necessitating further expansion and exploration.

When developing and optimizing a surgical strategy for DS, it is crucial to consider the clinical symptoms, general condition, and imaging presentation of the patient. While short-segment fixation and fusion have demonstrated considerable success in improving dysfunction and relieving pain, it is important to note that patients undergoing these procedures are typically elderly and susceptible to persistent back pain and accelerated degeneration of adjacent segments. Therefore, this study represents a significant contribution toward mitigating back pain and lowering the risk of adjacent segment degeneration in the elderly population.

## Supplementary Material

Supplementary material
