# Supplementary material for: Comparison of short-segment and long-segment fixation in treatment of degenerative scoliosis and analysis of factors associated with adjacent spondylolisthesis
Source: Open Med (Wars). 2024 Jun 21;19(1):20240983. doi: 10.1515/med-2024-0983 (PMC11193356; doi:10.1515/med-2024-0983)
Supplement: Supplementary material [file med-2024-0983-sm.pdf]

Supplementary material

Table S1: All patients clinical data

Patients		Pre operative	3 months post operative	6 months post operative	12 months post operative	Follow up
1 (Short segment fixation)	Clinical symptom	Chronic back pain	Minor improvement and inflammation	Moderate improvement and mild inflammation	Improved and slightly inflamed	Complete improvement and no inflammation
	Disease duration (Years)	12.8	—	—	—	—
	Cobb angle (°)	10.47	7.62	6.58	5.69	4.23
	Imaging information	Lumbar disc herniation and lumbar intervertebral spondylolisthesis of the relevant segments seen on CT and MRI	Minor improvement and inflammation	Moderate improvement and mild inflammation	Improved and slightly inflamed	Complete improvement and no inflammation
	VAS score	7.25	5.78	4.32	3.71	3.21
2 (Short segment fixation)	Clinical symptom	Presence of trauma	Minor improvement and inflammation	Moderate improvement and mild inflammation	Improved and slightly inflamed	Complete improvement and no inflammation
	Disease duration (Years)	9.2	—	—	—	—
	Cobb angle (°)	11.52	8.26	7.37	5.19	4.64
	Imaging information	Lumbar disc herniation and lumbar intervertebral spondylolisthesis of the relevant segments seen on CT and MRI	Minor improvement and inflammation	Moderate improvement and mild inflammation	Improved and slightly inflamed	Complete improvement and no inflammation
	VAS score	7.11	5.47	4.38	3.62	2.84
3 (Long segment fixation)	Clinical symptom	Soreness and pain in the lower back and legs recurring over a long period of time	Minor improvement and inflammation	Moderate improvement and mild inflammation	Improved and slightly inflamed	Complete improvement and no inflammation
	Disease duration (Years)	16.1	—	—	—	—
	Cobb angle (°)	13.03	10.38	7.62	5.74	4.94
	Imaging information	Degenerative lumbar disc changes, lumbar disc herniation,				

(Continued)

Table S1: *Continued*

Patients		Pre operative	3 months post operative	6 months post operative	12 months post operative	Follow up
		and lumbar intervertebral spondylolisthesis in the relevant segments can be seen on CT and MRI	Minor improvement and inflammation	Moderate improvement and mild inflammation	Improved and slightly inflamed	Complete improvement and no inflammation
	VAS score	7.07	5.78	4.35	3.42	2.83
4 (Short segment fixation)	Clinical symptom	Pain and numbness in the lower limbs or difficulty in walking, aggravated by standing or walking and relieved by lying down	Minor improvement and inflammation	Moderate improvement and mild inflammation	Improved and slightly inflamed	Complete improvement and no inflammation
	Disease duration (Years)	4.9	—	—	—	—
	Cobb angle (°)	10.23	7.45	6.67	5.24	4.87
	Imaging information	Lumbar intervertebral spondylolisthesis of the relevant segment seen on CT, MRI	Minor improvement and inflammation	Moderate improvement and mild inflammation	Improved and slightly inflamed	Complete improvement and no inflammation
	VAS score	7.31	5.70	4.37	3.65	2.89
5 (Short segment fixation)	Clinical symptom	Lumbago	Minor improvement and inflammation	Moderate improvement and mild inflammation	Improved and slightly inflamed	Complete improvement and no inflammation
	Disease duration (Years)	13.5	—	—	—	—
	Cobb angle (°)	11.12	8.36	6.75	5.58	4.04
	Imaging information	Lumbar intervertebral spinal stenosis of the relevant segment seen on CT, MRI	Minor improvement and inflammation	Moderate improvement and mild inflammation	Improved and slightly inflamed	Complete improvement and no inflammation
	VAS score	7.68	5.93	4.72	3.64	2.89
6 (Long segment fixation)	Clinical symptom	Pain and numbness in the lower limbs, aggravated by sitting, walking and exertion	Minor improvement and inflammation	Moderate improvement and mild inflammation	Improved and slightly inflamed	Complete improvement and no inflammation
	Disease duration (Years)	6.7	—	—	—	—
	Cobb angle (°)	10.85	8.73	6.46	5.37	4.06
	Imaging information	Degenerative changes of lumbar intervertebral discs in the relevant				

(Continued)

Table S1: *Continued*

Patients		Pre operative	3 months post operative	6 months post operative	12 months post operative	Follow up
		segments, lumbar intervertebral spondylolisthesis, as seen on CT, MRI	Minor improvement and inflammation	Moderate improvement and mild inflammation	Improved and slightly inflamed	Complete improvement and no inflammation
	VAS score	7.26	5.67	4.32	3.36	3.02
7 (Short segment fixation)	Clinical symptom	Intermittent claudication with marked neurogenic symptoms	Minor improvement and inflammation	Moderate improvement and mild inflammation	Improved and slightly inflamed	Complete improvement and no inflammation
	Disease duration (Years)	12.1	—	—	—	—
	Cobb angle (°)	12.25	8.47	7.34	6.12	4.72
	Imaging information	Lumbar disc herniation, lumbar intervertebral slip, lumbar intervertebral canal stenosis in the relevant segment seen on CT, MRI	Minor improvement and inflammation	Moderate improvement and mild inflammation	Improved and slightly inflamed	Complete improvement and no inflammation
	VAS score	7.57	5.89	4.34	3.41	2.92
8 (Short segment fixation)	Clinical symptom	Scoliosis deformity with corresponding pressure and percussion pain and radiating neurological symptoms in the lower limbs	Minor improvement and inflammation	Moderate improvement and mild inflammation	Improved and slightly inflamed	Complete improvement and no inflammation
	Disease duration (Years)	10.7	—	—	—	—
	Cobb angle (°)	10.68	7.52	6.63	6.56	4.82
	Imaging information	Lumbar disc herniation and lumbar intervertebral canal stenosis in the relevant segment seen on CT and MRI	Minor improvement and inflammation	Moderate improvement and mild inflammation	Improved and slightly inflamed	Complete improvement and no inflammation
	VAS score	7.56	5.91	4.87	3.72	3.14
9 (Long segment fixation)	Clinical symptom	Pathological reflexes are demonstrated by a straight leg raise test (+)	Minor improvement and inflammation	Moderate improvement and mild inflammation	Improved and slightly inflamed	Complete improvement and no inflammation
	Disease duration (Years)	18.3	—	—	—	—
	Cobb angle (°)	15.21	11.73	7.57	5.68	5.04

(Continued)

Table S1: *Continued*

Patients		Pre operative	3 months post operative	6 months post operative	12 months post operative	Follow up
	Imaging information	Lumbar degenerative disc changes, lumbar disc herniation, lumbar intervertebral slip, lumbar intervertebral canal stenosis in the relevant segments seen on CT, MRI	Minor improvement and inflammation	Moderate improvement and mild inflammation	Improved and slightly inflamed	Complete improvement and no inflammation
	VAS score	7.46	5.78	4.35	3.61	3.04
10 (Short segment fixation)	Clinical symptom	Pathological reflexes are demonstrated by a straight leg raise test (+)	Minor improvement and inflammation	Moderate improvement and mild inflammation	Improved and slightly inflamed	Complete improvement and no inflammation
	Disease duration (Years)	13.2	—	—	—	—
	Cobb angle (°)	11.51	8.64	7.32	6.75	4.17
	Imaging information	Lumbar intervertebral slippage and lumbar intervertebral stenosis of the relevant segments seen on CT and MRI	Minor improvement and inflammation	Moderate improvement and mild inflammation	Improved and slightly inflamed	Complete improvement and no inflammation
	VAS score	7.37	5.72	4.68	3.57	3.08
11 (Short segment fixation)	Clinical symptom	Presence of trauma	Minor improvement and inflammation	Moderate improvement and mild inflammation	Improved and slightly inflamed	Complete improvement and no inflammation
	Disease duration (Years)	14.5	—	—	—	—
	Cobb angle (°)	11.06	8.37	6.72	6.42	4.45
	Imaging information	Lumbar disc herniation, lumbar intervertebral slip, lumbar intervertebral canal stenosis in the relevant segment seen on CT, MRI	Minor improvement and inflammation	Moderate improvement and mild inflammation	Improved and slightly inflamed	Complete improvement and no inflammation
	VAS score	8.02	6.18	5.61	4.27	3.64
12 (Long segment fixation)	Clinical symptom	Intermittent claudication with marked neurogenic symptoms	Minor improvement and inflammation	Moderate improvement and mild inflammation	Improved and slightly inflamed	Complete improvement and no inflammation
	Disease duration (Years)	12.6	—	—	—	—

(Continued)

Table S1: *Continued*

Patients		Pre operative	3 months post operative	6 months post operative	12 months post operative	Follow up
	Cobb angle (°)	13.27	10.34	8.61	6.78	5.92
	Imaging information	Degenerative lumbar disc changes, lumbar disc herniation, and lumbar intervertebral spondylolisthesis in the relevant segments can be seen on CT and MRI	Minor improvement and inflammation	Moderate improvement and mild inflammation	Improved and slightly inflamed	Complete improvement and no inflammation
	VAS score	7.29	5.93	5.12	4.05	3.27
13 (Short segment fixation)	Clinical symptom	Scoliosis deformity with corresponding pressure and percussion pain and radiating neurological symptoms in the lower limbs	Minor improvement and inflammation	Moderate improvement and mild inflammation	Improved and slightly inflamed	Complete improvement and no inflammation
	Disease duration (Years)	11.9	—	—	—	—
	Cobb angle (°)	10.42	8.25	7.06	5.47	4.91
	Imaging information	Lumbar disc herniation and lumbar intervertebral canal stenosis in the relevant segment seen on CT and MRI	Minor improvement and inflammation	Moderate improvement and mild inflammation	Improved and slightly inflamed	Complete improvement and no inflammation
	VAS score	7.75	6.34	5.09	4.13	3.07
14 (Short segment fixation)	Clinical symptom	Lumbago	Minor improvement and inflammation	Moderate improvement and mild inflammation	Improved and slightly inflamed	Complete improvement and no inflammation
	Disease duration (Years)	10.5	—	—	—	—
	Cobb angle (°)	10.86	7.45	6.71	5.65	4.97
	Imaging information	Lumbar disc herniation and lumbar intervertebral spondylolisthesis of the relevant segments seen on CT and MRI	Minor improvement and inflammation	Moderate improvement and mild inflammation	Improved and slightly inflamed	Complete improvement and no inflammation
	VAS score	7.56	6.02	4.53	4.15	3.67
15 (Long segment fixation)	Clinical symptom	Soreness and pain in the lower back and legs recurring over a long period of time	Minor improvement and inflammation	Moderate improvement and mild inflammation	Improved and slightly inflamed	Complete improvement and no inflammation
		9.7	—	—	—	—

(Continued)

Table S1: *Continued*

Patients		Pre operative	3 months post operative	6 months post operative	12 months post operative	Follow up
	Disease duration (Years)					
	Cobb angle (°)	12.38	10.58	8.62	6.81	4.56
	Imaging information	Lumbar intervertebral spinal stenosis of the relevant segment seen on CT, MRI	Minor improvement and inflammation	Moderate improvement and mild inflammation	Improved and slightly inflamed	Complete improvement and no inflammation
	VAS score	7.45	5.87	4.92	3.86	3.08
16 (Short segment fixation)	Clinical symptom	Presence of trauma	Minor improvement and inflammation	Moderate improvement and mild inflammation	Improved and slightly inflamed	Complete improvement and no inflammation
	Disease duration (Years)	9.4	—	—	—	—
	Cobb angle (°)	11.12	8.51	7.74	5.81	4.26
	Imaging information	Degenerative changes of lumbar intervertebral discs in the relevant segments, lumbar intervertebral disc herniation visible under CT, MRI	Minor improvement and inflammation	Moderate improvement and mild inflammation	Improved and slightly inflamed	Complete improvement and no inflammation
	VAS score	7.56	5.83	4.39	3.47	3.01
17 (Short segment fixation)	Clinical symptom	Chronic back pain	Minor improvement and inflammation	Moderate improvement and mild inflammation	Improved and slightly inflamed	Complete improvement and no inflammation
	Disease duration (Years)	8.5	—	—	—	—
	Cobb angle (°)	11.23	8.43	7.53	5.78	4.99
	Imaging information	Degenerative changes of lumbar intervertebral discs in the relevant segments seen on CT, MRI	Minor improvement and inflammation	Moderate improvement and mild inflammation	Improved and slightly inflamed	Complete improvement and no inflammation
	VAS score	6.92	5.57	4.61	3.94	3.28
18 (Long segment fixation)	Clinical symptom	Chronic back pain	Minor improvement and inflammation	Moderate improvement and mild inflammation	Improved and slightly inflamed	Complete improvement and no inflammation
	Disease duration (Years)	8.4	—	—	—	—

(Continued)

Table S1: *Continued*

Patients		Pre operative	3 months post operative	6 months post operative	12 months post operative	Follow up
	Cobb angle (°)	14.12	10.46	8.51	6.97	5.05
	Imaging information	Lumbar disc herniation, lumbar intervertebral slip, lumbar intervertebral canal stenosis in the relevant segment seen on CT, MRI	Minor improvement and inflammation	Moderate improvement and mild inflammation	Improved and slightly inflamed	Complete improvement and no inflammation
	VAS score	7.19	6.25	5.36	4.72	3.97
19 (Short segment fixation)	Clinical symptom	Presence of trauma	Minor improvement and inflammation	Moderate improvement and mild inflammation	Improved and slightly inflamed	Complete improvement and no inflammation
	Disease duration (Years)	12.5	—	—	—	—
	Cobb angle (°)	11.37	8.26	6.82	5.24	4.62
	Imaging information	Degenerative changes of lumbar intervertebral discs in the relevant segments, lumbar intervertebral spondylolisthesis, as seen on CT, MRI	Minor improvement and inflammation	Moderate improvement and mild inflammation	Improved and slightly inflamed	Complete improvement and no inflammation
	VAS score	7.35	6.67	5.83	4.94	4.11
20 (Short segment fixation)	Clinical symptom	Pain and numbness in the lower limbs or difficulty in walking, aggravated by standing or walking and relieved by lying down	Minor improvement and inflammation	Moderate improvement and mild inflammation	Improved and slightly inflamed	Complete improvement and no inflammation
	Disease duration (Years)	13.4	—	—	—	—
	Cobb angle (°)	10.71	8.15	6.68	5.45	4.24
	Imaging information	Lumbar disc herniation and lumbar intervertebral canal stenosis in the relevant segment seen on CT and MRI	Minor improvement and inflammation	Moderate improvement and mild inflammation	Improved and slightly inflamed	Complete improvement and no inflammation
	VAS score	7.31	6.57	5.54	4.89	3.72
21 (Long segment fixation)	Clinical symptom	Lumbago	Minor improvement and inflammation	Moderate improvement and mild inflammation	Improved and slightly inflamed	Complete improvement and no inflammation
		14.3	—	—	—	—

(Continued)

Table S1: *Continued*

Patients		Pre operative	3 months post operative	6 months post operative	12 months post operative	Follow up
	Disease duration (Years)					
	Cobb angle (°)	12.76	9.25	7.47	6.12	5.63
	Imaging information	Lumbar degenerative disc changes, lumbar disc herniation, lumbar intervertebral slip, lumbar intervertebral canal stenosis in the relevant segments seen on CT, MRI	Minor improvement and inflammation	Moderate improvement and mild inflammation	Improved and slightly inflamed	Complete improvement and no inflammation
	VAS score	7.25	6.53	5.71	4.93	4.06
22 (Short segment fixation)	Clinical symptom	Lumbago	Minor improvement and inflammation	Moderate improvement and mild inflammation	Improved and slightly inflamed	Complete improvement and no inflammation
	Disease duration (Years)	15.6	—	—	—	—
	Cobb angle (°)	10.28	7.67	6.36	5.89	4.45
	Imaging information	Lumbar disc herniation, lumbar intervertebral slip, lumbar intervertebral canal stenosis in the relevant segment seen on CT, MRI	Minor improvement and inflammation	Moderate improvement and mild inflammation	Improved and slightly inflamed	Complete improvement and no inflammation
	VAS score	7.34	6.86	5.91	4.75	4.01
23 (Short segment fixation)	Clinical symptom	Soreness and pain in the lower back and legs recurring over a long period of time	Minor improvement and inflammation	Moderate improvement and mild inflammation	Improved and slightly inflamed	Complete improvement and no inflammation
	Disease duration (Years)	15.4	—	—	—	—
	Cobb angle (°)	10.71	8.04	6.56	5.22	4.18
	Imaging information	Lumbar intervertebral slippage and lumbar intervertebral stenosis of the relevant segments seen on CT and MRI	Minor improvement and inflammation	Moderate improvement and mild inflammation	Improved and slightly inflamed	Complete improvement and no inflammation
	VAS score	6.93	6.12	5.17	4.05	3.26
	Clinical symptom					

(Continued)

Table S1: *Continued*

Patients		Pre operative	3 months post operative	6 months post operative	12 months post operative	Follow up
24 (Long segment fixation)		Pain and numbness in the lower limbs, aggravated by sitting, walking and exertion	Minor improvement and inflammation	Moderate improvement and mild inflammation	Improved and slightly inflamed	Complete improvement and no inflammation
	Disease duration (Years)	16.0	—	—	—	—
	Cobb angle (°)	10.93	8.67	6.22	5.26	4.26
	Imaging information	Lumbar disc herniation and lumbar intervertebral spondylolisthesis of the relevant segments seen on CT and MRI	Minor improvement and inflammation	Moderate improvement and mild inflammation	Improved and slightly inflamed	Complete improvement and no inflammation
	VAS score	7.42	6.58	5.93	4.75	4.03
25 (Short segment fixation)	Clinical symptom	Intermittent claudication with marked neurogenic symptoms	Minor improvement and inflammation	Moderate improvement and mild inflammation	Improved and slightly inflamed	Complete improvement and no inflammation
	Disease duration (Years)	14.3	—	—	—	—
	Cobb angle (°)	11.57	8.21	7.26	5.38	4.01
	Imaging information	Degenerative lumbar disc changes, lumbar disc herniation, and lumbar intervertebral spondylolisthesis in the relevant segments can be seen on CT and MRI	Minor improvement and inflammation	Moderate improvement and mild inflammation	Improved and slightly inflamed	Complete improvement and no inflammation
	VAS score	7.08	6.14	5.29	4.23	3.65
26 (Short segment fixation)	Clinical symptom	Soreness and pain in the lower back and legs recurring over a long period of time	Minor improvement and inflammation	Moderate improvement and mild inflammation	Improved and slightly inflamed	Complete improvement and no inflammation
	Disease duration (Years)	13.7	—	—	—	—
	Cobb angle (°)	10.97	8.51	6.68	5.29	4.27
	Imaging information	Radiographs suggest a scoliotic deformity with a Cobb >10°, which may be associated with slippage or stenosis	Minor improvement and inflammation	Moderate improvement and mild inflammation	Improved and slightly inflamed	Complete improvement and no inflammation
	VAS score	7.04	6.13	5.47	4.26	3.53

(Continued)

Table S1: *Continued*

Patients		Pre operative	3 months post operative	6 months post operative	12 months post operative	Follow up
27 (Long segment fixation)	Clinical symptom	Chronic back pain	Minor improvement and inflammation	Moderate improvement and mild inflammation	Improved and slightly inflamed	Complete improvement and no inflammation
	Disease duration (Years)	12.5	—	—	—	—
	Cobb angle (°)	11.43	7.37	6.35	5.22	4.57
	Imaging information	Degenerative changes of lumbar intervertebral discs and lumbar intervertebral spinal stenosis in the relevant segments were seen on CT and MRI	Minor improvement and inflammation	Moderate improvement and mild inflammation	Improved and slightly inflamed	Complete improvement and no inflammation
	VAS score	7.36	6.12	5.47	4.83	3.68
28 (Short segment fixation)	Clinical symptom	Presence of trauma	Minor improvement and inflammation	Moderate improvement and mild inflammation	Improved and slightly inflamed	Complete improvement and no inflammation
	Disease duration (Years)	11.4	—	—	—	—
	Cobb angle (°)	10.92	8.54	7.23	5.18	4.28
	Imaging information	Degenerative changes of lumbar intervertebral discs in the relevant segments seen on CT, MRI	Minor improvement and inflammation	Moderate improvement and mild inflammation	Improved and slightly inflamed	Complete improvement and no inflammation
	VAS score	7.25	6.37	5.53	4.93	3.64
29 (Short segment fixation)	Clinical symptom	Intermittent claudication with marked neurogenic symptoms	Minor improvement and inflammation	Moderate improvement and mild inflammation	Improved and slightly inflamed	Complete improvement and no inflammation
	Disease duration (Years)	14.7	—	—	—	—
	Cobb angle (°)	10.91	7.85	6.26	5.78	4.54
	Imaging information	Lumbar intervertebral slippage and lumbar intervertebral stenosis of the relevant segments seen on CT and MRI	Minor improvement and inflammation	Moderate improvement and mild inflammation	Improved and slightly inflamed	Complete improvement and no inflammation
	VAS score	7.47	6.31	5.76	4.84	4.12
	Clinical symptom	Scoliosis deformity with corresponding pressure and				

(Continued)

Table S1: *Continued*

Patients		Pre operative	3 months post operative	6 months post operative	12 months post operative	Follow up
30 (Long segment fixation)		percussion pain and radiating neurological symptoms in the lower limbs	Minor improvement and inflammation	Moderate improvement and mild inflammation	Improved and slightly inflamed	Complete improvement and no inflammation
	Disease duration (Years)	15.4	—	—	—	—
	Cobb angle (°)	12.67	10.85	7.54	6.26	4.78
	Imaging information	Lumbar degenerative disc changes, lumbar disc herniation, lumbar intervertebral slip, lumbar intervertebral canal stenosis in the relevant segments seen on CT, MRI	Minor improvement and inflammation	Moderate improvement and mild inflammation	Improved and slightly inflamed	Complete improvement and no inflammation
	VAS score	6.93	6.04	5.17	4.25	2.91
31 (Short segment fixation)	Clinical symptom	Chronic back pain	Minor improvement and inflammation	Moderate improvement and mild inflammation	Improved and slightly inflamed	Complete improvement and no inflammation
	Disease duration (Years)	10.9	—	—	—	—
	Cobb angle (°)	11.12	8.05	6.71	5.84	4.72
	Imaging information	Radiographically suggestive of scoliotic deformity, Cobb >10°, with degenerative changes of the vertebral body, synovial hyperplasia, and intervertebral space	Minor improvement and inflammation	Moderate improvement and mild inflammation	Improved and slightly inflamed	Complete improvement and no inflammation
	VAS score	7.24	6.32	5.21	4.38	3.27
32 (Short segment fixation)	Clinical symptom	Presence of trauma	Minor improvement and inflammation	Moderate improvement and mild inflammation	Improved and slightly inflamed	Complete improvement and no inflammation
	Disease duration (Years)	11.6	—	—	—	—
	Cobb angle (°)	10.75	8.52	6.28	5.46	4.82
	Imaging information	Degenerative changes of lumbar intervertebral discs in the relevant segments, lumbar intervertebral	Minor improvement and inflammation	Moderate improvement and mild inflammation	Improved and slightly inflamed	Complete improvement and no inflammation

(Continued)

Table S1: *Continued*

Patients		Pre operative	3 months post operative	6 months post operative	12 months post operative	Follow up
		spondylolisthesis, as seen on CT, MRI				
	VAS score	7.35	6.47	5.38	4.51	3.93
33 (Long segment fixation)	Clinical symptom	Lumbago	Minor improvement and inflammation	Moderate improvement and mild inflammation	Improved and slightly inflamed	Complete improvement and no inflammation
	Disease duration (Years)	11.2	—	—	—	—
	Cobb angle (°)	10.58	8.12	6.25	5.79	4.74
	Imaging information	Lumbar disc herniation and lumbar intervertebral canal stenosis in the relevant segment seen on CT and MRI	Minor improvement and inflammation	Moderate improvement and mild inflammation	Improved and slightly inflamed	Complete improvement and no inflammation
	VAS score	7.35	6.41	5.37	4.43	3.55
34 (Short segment fixation)	Clinical symptom	Pain and numbness in the lower limbs, aggravated by sitting, walking and exertion	Minor improvement and inflammation	Moderate improvement and mild inflammation	Improved and slightly inflamed	Complete improvement and no inflammation
	Disease duration (Years)	11.7	—	—	—	—
	Cobb angle (°)	10.57	8.46	7.12	5.73	4.78
	Imaging information	Lumbar disc herniation and lumbar intervertebral spondylolisthesis of the relevant segments seen on CT and MRI	Minor improvement and inflammation	Moderate improvement and mild inflammation	Improved and slightly inflamed	Complete improvement and no inflammation
	VAS score	7.46	6.67	5.82	4.91	4.36
35 (Short segment fixation)	Clinical symptom	Intermittent claudication with marked neurogenic symptoms	Minor improvement and inflammation	Moderate improvement and mild inflammation	Improved and slightly inflamed	Complete improvement and no inflammation
	Disease duration (Years)	11.9	—	—	—	—
	Cobb angle (°)	11.12	8.68	6.56	5.59	4.91
	Imaging information	Lumbar intervertebral slippage and lumbar intervertebral stenosis of the relevant segments seen on CT and MRI	Minor improvement and inflammation	Moderate improvement and mild inflammation	Improved and slightly inflamed	Complete improvement and no inflammation

(Continued)

Table S1: *Continued*

Patients		Pre operative	3 months post operative	6 months post operative	12 months post operative	Follow up
	VAS score	7.31	6.56	5.72	4.71	3.92
36 (Long segment fixation)	Clinical symptom	Pathological reflexes are demonstrated by a straight leg raise test (+)	Minor improvement and inflammation	Moderate improvement and mild inflammation	Improved and slightly inflamed	Complete improvement and no inflammation
	Disease duration (Years)	13.2	—	—	—	—
	Cobb angle (°)	10.73	9.06	7.45	6.63	5.48
	Imaging information	Lumbar disc herniation, lumbar intervertebral slip, lumbar intervertebral canal stenosis in the relevant segment seen on CT, MRI	Minor improvement and inflammation	Moderate improvement and mild inflammation	Improved and slightly inflamed	Complete improvement and no inflammation
	VAS score	7.22	6.57	5.85	4.32	3.75
37 (Short segment fixation)	Clinical symptom	Soreness and pain in the lower back and legs recurring over a long period of time	Minor improvement and inflammation	Moderate improvement and mild inflammation	Improved and slightly inflamed	Complete improvement and no inflammation
	Disease duration (Years)	14.2	—	—	—	—
	Cobb angle (°)	10.95	8.04	6.71	4.92	4.06
	Imaging information	Lumbar disc herniation of the relevant segment seen on CT, MRI	Minor improvement and inflammation	Moderate improvement and mild inflammation	Improved and slightly inflamed	Complete improvement and no inflammation
	VAS score	7.19	6.24	5.45	4.12	3.41
38 (Short segment fixation)	Clinical symptom	Presence of trauma	Minor improvement and inflammation	Moderate improvement and mild inflammation	Improved and slightly inflamed	Complete improvement and no inflammation
	Disease duration (Years)	12.9	—	—	—	—
	Cobb angle (°)	11.35	8.71	7.56	5.87	4.28
	Imaging information	Degenerative changes of lumbar intervertebral discs and lumbar intervertebral spinal stenosis in the relevant segments were seen on CT and MRI	Minor improvement and inflammation	Moderate improvement and mild inflammation	Improved and slightly inflamed	Complete improvement and no inflammation
	VAS score	7.32	6.34	5.71	4.95	4.16

(Continued)

Table S1: *Continued*

Patients		Pre operative	3 months post operative	6 months post operative	12 months post operative	Follow up
39 (Long segment fixation)	Clinical symptom	Chronic back pain	Minor improvement and inflammation	Moderate improvement and mild inflammation	Improved and slightly inflamed	Complete improvement and no inflammation
	Disease duration (Years)	9.5	—	—	—	—
	Cobb angle (°)	12.85	9.76	7.92	6.63	5.78
	Imaging information	Lumbar degenerative disc changes, lumbar disc herniation, lumbar intervertebral slip, lumbar intervertebral canal stenosis in the relevant segments seen on CT, MRI	Minor improvement and inflammation	Moderate improvement and mild inflammation	Improved and slightly inflamed	Complete improvement and no inflammation
	VAS score	6.84	6.12	5.32	4.09	3.36
40 (Short segment fixation)	Clinical symptom	Pathological reflexes are demonstrated by a straight leg raise test (+)	Minor improvement and inflammation	Moderate improvement and mild inflammation	Improved and slightly inflamed	Complete improvement and no inflammation
	Disease duration (Years)	10.1	—	—	—	—
	Cobb angle (°)	11.14	8.62	7.48	5.56	4.93
	Imaging information	Lumbar intervertebral slippage and lumbar intervertebral stenosis of the relevant segments seen on CT and MRI	Minor improvement and inflammation	Moderate improvement and mild inflammation	Improved and slightly inflamed	Complete improvement and no inflammation
	VAS score	7.35	6.84	5.19	4.45	4.02
41 (Short segment fixation)	Clinical symptom	Pain and numbness in the lower limbs, aggravated by sitting, walking and exertion	Minor improvement and inflammation	Moderate improvement and mild inflammation	Improved and slightly inflamed	Complete improvement and no inflammation
	Disease duration (Years)	9.7	—	—	—	—
	Cobb angle (°)	11.28	8.26	7.85	5.47	4.91
	Imaging information	Lumbar disc herniation and lumbar intervertebral spondylolisthesis of the relevant segments seen on CT and MRI	Minor improvement and inflammation	Moderate improvement and mild inflammation	Improved and slightly inflamed	Complete improvement and no inflammation
	VAS score	7.08	6.25	5.41	4.73	3.91

(Continued)

Table S1: *Continued*

Patients		Pre operative	3 months post operative	6 months post operative	12 months post operative	Follow up
42 (Long segment fixation)	Clinical symptom	Intermittent claudication with marked neurogenic symptoms	Minor improvement and inflammation	Moderate improvement and mild inflammation	Improved and slightly inflamed	Complete improvement and no inflammation
	Disease duration (Years)	9.2	—	—	—	—
	Cobb angle (°)	12.67	10.86	7.92	6.38	5.91
	Imaging information	Lumbar disc herniation and lumbar intervertebral canal stenosis in the relevant segment seen on CT and MRI	Minor improvement and inflammation	Moderate improvement and mild inflammation	Improved and slightly inflamed	Complete improvement and no inflammation
	VAS score	7.27	6.45	5.67	4.81	3.95
43 (Short segment fixation)	Clinical symptom	Soreness and pain in the lower back and legs recurring over a long period of time	Minor improvement and inflammation	Moderate improvement and mild inflammation	Improved and slightly inflamed	Complete improvement and no inflammation
	Disease duration (Years)	8.7	—	—	—	—
	Cobb angle (°)	11.58	9.05	7.47	5.96	4.85
	Imaging information	Lumbar disc herniation, lumbar intervertebral slip, lumbar intervertebral canal stenosis in the relevant segment seen on CT, MRI	Minor improvement and inflammation	Moderate improvement and mild inflammation	Improved and slightly inflamed	Complete improvement and no inflammation
	VAS score	7.02	6.45	5.61	4.92	4.33
44 (Short segment fixation)	Clinical symptom	Presence of trauma	Minor improvement and inflammation	Moderate improvement and mild inflammation	Improved and slightly inflamed	Complete improvement and no inflammation
	Disease duration (Years)	11.2	—	—	—	—
	Cobb angle (°)	10.75	7.45	6.62	5.81	3.97
	Imaging information	Degenerative lumbar disc changes, lumbar disc herniation, and lumbar intervertebral spondylolisthesis in the relevant segments can be seen on CT and MRI	Minor improvement and inflammation	Moderate improvement and mild inflammation	Improved and slightly inflamed	Complete improvement and no inflammation

(Continued)

Table S1: *Continued*

Patients		Pre operative	3 months post operative	6 months post operative	12 months post operative	Follow up
	VAS score	7.47	6.29	5.61	4.78	3.93
45 (Long segment fixation)	Clinical symptom	Chronic back pain	Minor improvement and inflammation	Moderate improvement and mild inflammation	Improved and slightly inflamed	Complete improvement and no inflammation
	Disease duration (Years)	14.0	—	—	—	—
	Cobb angle (°)	12.67	10.08	7.54	6.46	5.74
	Imaging information	Degenerative changes of lumbar intervertebral discs and lumbar intervertebral spinal stenosis in the relevant segments were seen on CT and MRI	Minor improvement and inflammation	Moderate improvement and mild inflammation	Improved and slightly inflamed	Complete improvement and no inflammation
	VAS score	7.31	6.56	5.78	4.52	3.84
46 (Short segment fixation)	Clinical symptom	Pain and numbness in the lower limbs, aggravated by sitting, walking and exertion	Minor improvement and inflammation	Moderate improvement and mild inflammation	Improved and slightly inflamed	Complete improvement and no inflammation
	Disease duration (Years)	13.2	—	—	—	—
	Cobb angle (°)	10.28	8.02	6.71	5.44	3.95
	Imaging information	Degenerative changes of lumbar intervertebral discs in the relevant segments seen on CT, MRI	Minor improvement and inflammation	Moderate improvement and mild inflammation	Improved and slightly inflamed	Complete improvement and no inflammation
	VAS score	7.03	6.57	5.16	4.27	3.67
47 (Short segment fixation)	Clinical symptom	Intermittent claudication with marked neurogenic symptoms	Minor improvement and inflammation	Moderate improvement and mild inflammation	Improved and slightly inflamed	Complete improvement and no inflammation
	Disease duration (Years)	13.4	—	—	—	—
	Cobb angle (°)	10.94	7.63	6.37	5.61	3.89
	Imaging information	Lumbar degenerative disc changes, lumbar disc herniation, lumbar intervertebral slip, lumbar intervertebral canal stenosis in the relevant segments seen on CT, MRI	Minor improvement and inflammation	Moderate improvement and mild inflammation	Improved and slightly inflamed	Complete improvement and no inflammation

(Continued)

Table S1: *Continued*

Patients		Pre operative	3 months post operative	6 months post operative	12 months post operative	Follow up
48 (Long segment fixation)	VAS score	7.36	6.47	5.91	4.85	4.14
	Clinical symptom	Pathological reflexes are demonstrated by a straight leg raise test (+)	Minor improvement and inflammation	Moderate improvement and mild inflammation	Improved and slightly inflamed	Complete improvement and no inflammation
	Disease duration (Years)	12.6	—	—	—	—
	Cobb angle (°)	12.67	10.05	7.52	5.86	5.06
	Imaging information	Degenerative changes of lumbar intervertebral discs in the relevant segments, lumbar intervertebral disc herniation visible under CT, MRI	Minor improvement and inflammation	Moderate improvement and mild inflammation	Improved and slightly inflamed	Complete improvement and no inflammation
49 (Short segment fixation)	VAS score	7.46	6.95	5.72	4.61	4.07
	Clinical symptom	Pain and numbness in the lower limbs or difficulty in walking, aggravated by standing or walking and relieved by lying down	Minor improvement and inflammation	Moderate improvement and mild inflammation	Improved and slightly inflamed	Complete improvement and no inflammation
	Disease duration (Years)	13.6	—	—	—	—
	Cobb angle (°)	10.62	8.08	7.12	5.48	4.27
	Imaging information	Lumbar disc herniation, lumbar intervertebral slip, lumbar intervertebral canal stenosis in the relevant segment seen on CT, MRI	Minor improvement and inflammation	Moderate improvement and mild inflammation	Improved and slightly inflamed	Complete improvement and no inflammation
50 (Short segment fixation)	VAS score	7.08	6.47	5.93	4.58	3.12
	Clinical symptom	Pathological reflexes are demonstrated by a straight leg raise test (+)	Minor improvement and inflammation	Moderate improvement and mild inflammation	Improved and slightly inflamed	Complete improvement and no inflammation
	Disease duration (Years)	12.6	—	—	—	—
	Cobb angle (°)	10.37	7.92	7.14	5.32	3.85
	Imaging information	Lumbar disc herniation of the relevant segment seen on CT, MRI				

(Continued)

Table S1: *Continued*

Patients		Pre operative	3 months post operative	6 months post operative	12 months post operative	Follow up
			Minor improvement and inflammation	Moderate improvement and mild inflammation	Improved and slightly inflamed	Complete improvement and no inflammation
	VAS score	8.08	6.74	5.93	4.79	3.91
51 (Long segment fixation)	Clinical symptom	Soreness and pain in the lower back and legs recurring over a long period of time	Minor improvement and inflammation	Moderate improvement and mild inflammation	Improved and slightly inflamed	Complete improvement and no inflammation
	Disease duration (Years)	13.0	—	—	—	—
	Cobb angle (°)	12.27	9.64	7.35	5.91	4.56
	Imaging information	Lumbar intervertebral spondylolisthesis of the relevant segment seen on CT, MRI	Minor improvement and inflammation	Moderate improvement and mild inflammation	Improved and slightly inflamed	Complete improvement and no inflammation
	VAS score	7.78	6.45	5.42	4.95	4.19
52 (Short segment fixation)	Clinical symptom	Presence of trauma	Minor improvement and inflammation	Moderate improvement and mild inflammation	Improved and slightly inflamed	Complete improvement and no inflammation
	Disease duration (Years)	11.5	—	—	—	—
	Cobb angle (°)	10.95	7.81	7.62	5.78	4.67
	Imaging information	Lumbar disc herniation and lumbar intervertebral canal stenosis in the relevant segment seen on CT and MRI	Minor improvement and inflammation	Moderate improvement and mild inflammation	Improved and slightly inflamed	Complete improvement and no inflammation
	VAS score	7.91	6.74	5.62	4.27	4.35
53 (Short segment fixation)	Clinical symptom	Soreness and pain in the lower back and legs recurring over a long period of time	Minor improvement and inflammation	Moderate improvement and mild inflammation	Improved and slightly inflamed	Complete improvement and no inflammation
	Disease duration (Years)	11.2	—	—	—	—
	Cobb angle (°)	11.12	8.06	6.71	5.36	4.24
	Imaging information	Lumbar disc herniation of the relevant segment seen on CT, MRI	Minor improvement and inflammation	Moderate improvement and mild inflammation	Improved and slightly inflamed	Complete improvement and no inflammation
	VAS score	7.83	6.92	5.78	4.35	4.11

(Continued)

Table S1: *Continued*

Patients		Pre operative	3 months post operative	6 months post operative	12 months post operative	Follow up
54 (Long segment fixation)	Clinical symptom	Pain and numbness in the lower limbs, aggravated by sitting, walking and exertion	Minor improvement and inflammation	Moderate improvement and mild inflammation	Improved and slightly inflamed	Complete improvement and no inflammation
	Disease duration (Years)	11.7	—	—	—	—
	Cobb angle (°)	12.38	10.27	9.06	7.28	5.67
	Imaging information	Lumbar degenerative disc changes, lumbar disc herniation, lumbar intervertebral slip, lumbar intervertebral canal stenosis in the relevant segments seen on CT, MRI	Minor improvement and inflammation	Moderate improvement and mild inflammation	Improved and slightly inflamed	Complete improvement and no inflammation
	VAS score	7.76	6.81	5.64	4.74	4.20
55 (Short segment fixation)	Clinical symptom	Intermittent claudication with marked neurogenic symptoms	Minor improvement and inflammation	Moderate improvement and mild inflammation	Improved and slightly inflamed	Complete improvement and no inflammation
	Disease duration (Years)	9.6	—	—	—	—
	Cobb angle (°)	11.62	8.43	7.48	5.06	4.31
	Imaging information	Lumbar intervertebral spondylolisthesis of the relevant segment seen on CT, MRI	Minor improvement and inflammation	Moderate improvement and mild inflammation	Improved and slightly inflamed	Complete improvement and no inflammation
	VAS score	7.85	6.91	5.63	4.82	4.14
56 (Short segment fixation)	Clinical symptom	Pathological reflexes are demonstrated by a straight leg raise test (+)	Minor improvement and inflammation	Moderate improvement and mild inflammation	Improved and slightly inflamed	Complete improvement and no inflammation
	Disease duration (Years)	9.9	—	—	—	—
	Cobb angle (°)	11.18	8.34	7.29	5.21	4.84
	Imaging information	Lumbar disc herniation and lumbar intervertebral spondylolisthesis of the relevant segments seen on CT and MRI	Minor improvement and inflammation	Moderate improvement and mild inflammation	Improved and slightly inflamed	Complete improvement and no inflammation
	VAS score	7.51	6.35	5.39	4.24	3.73

(Continued)

Table S1: *Continued*

Patients		Pre operative	3 months post operative	6 months post operative	12 months post operative	Follow up
57 (Long segment fixation)	Clinical symptom	Pain and numbness in the lower limbs, aggravated by sitting, walking and exertion	Minor improvement and inflammation	Moderate improvement and mild inflammation	Improved and slightly inflamed	Complete improvement and no inflammation
	Disease duration (Years)	9.5	—	—	—	—
	Cobb angle (°)	10.79	8.67	6.35	4.78	4.14
	Imaging information	Lumbar intervertebral slippage and lumbar intervertebral stenosis of the relevant segments seen on CT and MRI	Minor improvement and inflammation	Moderate improvement and mild inflammation	Improved and slightly inflamed	Complete improvement and no inflammation
	VAS score	7.62	6.54	5.22	4.38	3.87
58 (Short segment fixation)	Clinical symptom	Soreness and pain in the lower back and legs recurring over a long period of time	Minor improvement and inflammation	Moderate improvement and mild inflammation	Improved and slightly inflamed	Complete improvement and no inflammation
	Disease duration (Years)	10.3	—	—	—	—
	Cobb angle (°)	11.06	7.84	7.22	5.37	3.95
	Imaging information	Lumbar disc herniation, lumbar intervertebral slip, lumbar intervertebral canal stenosis in the relevant segment seen on CT, MRI	Minor improvement and inflammation	Moderate improvement and mild inflammation	Improved and slightly inflamed	Complete improvement and no inflammation
	VAS score	7.84	6.92	5.67	4.58	4.13
59 (Short segment fixation)	Clinical symptom	Chronic back pain	Minor improvement and inflammation	Moderate improvement and mild inflammation	Improved and slightly inflamed	Complete improvement and no inflammation
	Disease duration (Years)	11.7	—	—	—	—
	Cobb angle (°)	11.57	8.67	6.75	5.04	3.92
	Imaging information	Degenerative lumbar disc changes, lumbar disc herniation, and lumbar intervertebral spondylolisthesis in the relevant segments can be seen on CT and MRI	Minor improvement and inflammation	Moderate improvement and mild inflammation	Improved and slightly inflamed	Complete improvement and no inflammation

(Continued)

Table S1: *Continued*

Patients		Pre operative	3 months post operative	6 months post operative	12 months post operative	Follow up
60 (Long segment fixation)	VAS score	7.36	6.73	5.52	4.54	3.94
	Clinical symptom	Pain and numbness in the lower limbs, aggravated by sitting, walking and exertion	Minor improvement and inflammation	Moderate improvement and mild inflammation	Improved and slightly inflamed	Complete improvement and no inflammation
	Disease duration (Years)	12.1	—	—	—	—
	Cobb angle (°)	10.85	8.94	7.67	5.06	4.73
	Imaging information	Radiographically suggestive of scoliotic deformity, Cobb >10°, with degenerative changes of the vertebral body, synovial hyperplasia, and intervertebral space	Minor improvement and inflammation	Moderate improvement and mild inflammation	Improved and slightly inflamed	Complete improvement and no inflammation
61 (Short segment fixation)	VAS score	7.57	6.92	5.45	4.28	3.41
	Clinical symptom	Pathological reflexes are demonstrated by a straight leg raise test (+)	Minor improvement and inflammation	Moderate improvement and mild inflammation	Improved and slightly inflamed	Complete improvement and no inflammation
	Disease duration (Years)	13.3	—	—	—	—
	Cobb angle (°)	11.73	7.91	7.14	5.18	4.23
	Imaging information	Degenerative changes of lumbar intervertebral discs and lumbar intervertebral spinal stenosis in the relevant segments were seen on CT and MRI	Minor improvement and inflammation	Moderate improvement and mild inflammation	Improved and slightly inflamed	Complete improvement and no inflammation
62 (Short segment fixation)	VAS score	7.65	6.92	5.47	4.26	4.65
	Clinical symptom	Pain and numbness in the lower limbs, aggravated by sitting, walking and exertion	Minor improvement and inflammation	Moderate improvement and mild inflammation	Improved and slightly inflamed	Complete improvement and no inflammation
	Disease duration (Years)	13.9	—	—	—	—
	Cobb angle (°)	11.45	8.67	7.35	5.32	3.92
	Imaging information	Lumbar disc herniation of the relevant segment seen on CT, MRI	Minor improvement and inflammation	Moderate improvement and mild inflammation	Improved and slightly inflamed	Complete improvement and no inflammation

(Continued)

Table S1: *Continued*

Patients		Pre operative	3 months post operative	6 months post operative	12 months post operative	Follow up
	VAS score	7.41	6.55	5.73	4.92	4.48
63 (Long segment fixation)	Clinical symptom	Soreness and pain in the lower back and legs recurring over a long period of time	Minor improvement and inflammation	Moderate improvement and mild inflammation	Improved and slightly inflamed	Complete improvement and no inflammation
	Disease duration (Years)	12.6	—	—	—	—
	Cobb angle (°)	10.82	9.34	7.57	6.39	6.13
	Imaging information	Lumbar disc herniation and lumbar intervertebral spondylolisthesis of the relevant segments seen on CT and MRI	Minor improvement and inflammation	Moderate improvement and mild inflammation	Improved and slightly inflamed	Complete improvement and no inflammation
	VAS score	7.28	6.93	5.71	4.64	4.13
64 (Short segment fixation)	Clinical symptom	Pain and numbness in the lower limbs, aggravated by sitting, walking and exertion	Minor improvement and inflammation	Moderate improvement and mild inflammation	Improved and slightly inflamed	Complete improvement and no inflammation
	Disease duration (Years)	12.3	—	—	—	—
	Cobb angle (°)	11.05	8.38	7.47	4.93	4.08
	Imaging information	Lumbar disc herniation and lumbar intervertebral canal stenosis in the relevant segment seen on CT and MRI	Minor improvement and inflammation	Moderate improvement and mild inflammation	Improved and slightly inflamed	Complete improvement and no inflammation
	VAS score	7.35	6.37	5.64	4.91	4.23
65 (Short segment fixation)	Clinical symptom	Intermittent claudication with marked neurogenic symptoms	Minor improvement and inflammation	Moderate improvement and mild inflammation	Improved and slightly inflamed	Complete improvement and no inflammation
	Disease duration (Years)	13.1	—	—	—	—
	Cobb angle (°)	11.75	7.92	7.08	5.47	4.56
	Imaging information	Lumbar intervertebral slippage and lumbar intervertebral stenosis of the relevant segments seen on CT and MRI	Minor improvement and inflammation	Moderate improvement and mild inflammation	Improved and slightly inflamed	Complete improvement and no inflammation
	VAS score	7.24	6.62	5.71	4.85	4.34

(Continued)

Table S1: *Continued*

Patients		Pre operative	3 months post operative	6 months post operative	12 months post operative	Follow up
66 (Long segment fixation)	Clinical symptom	Soreness and pain in the lower back and legs recurring over a long period of time	Minor improvement and inflammation	Moderate improvement and mild inflammation	Improved and slightly inflamed	Complete improvement and no inflammation
	Disease duration (Years)	14.5	—	—	—	—
	Cobb angle (°)	11.63	10.93	8.15	6.84	6.06
	Imaging information	Lumbar disc herniation, lumbar intervertebral slip, lumbar intervertebral canal stenosis in the relevant segment seen on CT, MRI	Minor improvement and inflammation	Moderate improvement and mild inflammation	Improved and slightly inflamed	Complete improvement and no inflammation
	VAS score	7.82	6.97	5.41	4.62	4.08
67 (Short segment fixation)	Clinical symptom	Chronic back pain	Minor improvement and inflammation	Moderate improvement and mild inflammation	Improved and slightly inflamed	Complete improvement and no inflammation
	Disease duration (Years)	15.2	—	—	—	—
	Cobb angle (°)	11.67	8.43	7.42	5.15	3.59
	Imaging information	Lumbar degenerative disc changes, lumbar disc herniation, lumbar intervertebral slip, lumbar intervertebral canal stenosis in the relevant segments seen on CT, MRI	Minor improvement and inflammation	Moderate improvement and mild inflammation	Improved and slightly inflamed	Complete improvement and no inflammation
	VAS score	7.54	6.82	5.36	4.53	4.09
68 (Short segment fixation)	Clinical symptom	Presence of trauma	Minor improvement and inflammation	Moderate improvement and mild inflammation	Improved and slightly inflamed	Complete improvement and no inflammation
	Disease duration (Years)	14.2	—	—	—	—
	Cobb angle (°)	11.37	7.93	6.74	5.45	3.87
	Imaging information	Degenerative changes of lumbar intervertebral discs in the relevant segments, lumbar intervertebral	Minor improvement and inflammation	Moderate improvement and mild inflammation	Improved and slightly inflamed	Complete improvement and no inflammation

(Continued)

Table S1: *Continued*

Patients		Pre operative	3 months post operative	6 months post operative	12 months post operative	Follow up
		disc herniation visible under CT, MRI				
	VAS score	7.84	6.93	5.71	4.92	4.48
69 (Long segment fixation)	Clinical symptom	Pain and numbness in the lower limbs, aggravated by sitting, walking and exertion	Minor improvement and inflammation	Moderate improvement and mild inflammation	Improved and slightly inflamed	Complete improvement and no inflammation
	Disease duration (Years)	12.4	—	—	—	—
	Cobb angle (°)	11.45	10.67	8.15	6.32	5.92
	Imaging information	Degenerative changes of lumbar intervertebral discs in the relevant segments seen on CT, MRI	Minor improvement and inflammation	Moderate improvement and mild inflammation	Improved and slightly inflamed	Complete improvement and no inflammation
	VAS score	7.35	6.17	5.03	4.26	3.19
70 (Short segment fixation)	Clinical symptom	Intermittent claudication with marked neurogenic symptoms	Minor improvement and inflammation	Moderate improvement and mild inflammation	Improved and slightly inflamed	Complete improvement and no inflammation
	Disease duration (Years)	12.2	—	—	—	—
	Cobb angle (°)	11.14	8.08	7.23	5.57	3.85
	Imaging information	Lumbar disc herniation and lumbar intervertebral canal stenosis in the relevant segment seen on CT and MRI	Minor improvement and inflammation	Moderate improvement and mild inflammation	Improved and slightly inflamed	Complete improvement and no inflammation
	VAS score	7.45	6.73	5.49	4.28	3.81
71 (Short segment fixation)	Clinical symptom	Pain and numbness in the lower limbs, aggravated by sitting, walking and exertion	Minor improvement and inflammation	Moderate improvement and mild inflammation	Improved and slightly inflamed	Complete improvement and no inflammation
	Disease duration (Years)	13.1	—	—	—	—
	Cobb angle (°)	11.68	7.73	7.05	5.12	3.64
	Imaging information	Degenerative changes of lumbar intervertebral discs and lumbar intervertebral spinal stenosis in the relevant segments were seen on CT and MRI	Minor improvement and inflammation	Moderate improvement and mild inflammation	Improved and slightly inflamed	Complete improvement and no inflammation

(Continued)

Table S1: *Continued*

Patients		Pre operative	3 months post operative	6 months post operative	12 months post operative	Follow up
	VAS score	7.54	6.96	5.72	4.91	4.56
72 (Long segment fixation)	Clinical symptom	Presence of trauma	Minor improvement and inflammation	Moderate improvement and mild inflammation	Improved and slightly inflamed	Complete improvement and no inflammation
	Disease duration (Years)	13.7	—	—	—	—
	Cobb angle (°)	11.39	10.71	8.47	7.35	6.48
	Imaging information	Degenerative changes of lumbar intervertebral discs in the relevant segments, lumbar intervertebral spondylolisthesis, as seen on CT, MRI	Minor improvement and inflammation	Moderate improvement and mild inflammation	Improved and slightly inflamed	Complete improvement and no inflammation
	VAS score	7.84	6.17	5.02	4.23	3.47
73 (Short segment fixation)	Clinical symptom	Chronic back pain	Minor improvement and inflammation	Moderate improvement and mild inflammation	Improved and slightly inflamed	Complete improvement and no inflammation
	Disease duration (Years)	13.3	—	—	—	—
	Cobb angle (°)	11.63	7.92	7.21	5.34	4.28
	Imaging information	Degenerative lumbar disc changes, lumbar disc herniation, and lumbar intervertebral spondylolisthesis in the relevant segments can be seen on CT and MRI	Minor improvement and inflammation	Moderate improvement and mild inflammation	Improved and slightly inflamed	Complete improvement and no inflammation
	VAS score	7.45	6.93	5.71	4.82	4.34
74 (Short segment fixation)	Clinical symptom	Pain and numbness in the lower limbs or difficulty in walking, aggravated by standing or walking and relieved by lying down	Minor improvement and inflammation	Moderate improvement and mild inflammation	Improved and slightly inflamed	Complete improvement and no inflammation
	Disease duration (Years)	14.2	—	—	—	—
	Cobb angle (°)	11.74	7.68	6.49	5.31	3.85

(Continued)

Table S1: *Continued*

Patients		Pre operative	3 months post operative	6 months post operative	12 months post operative	Follow up
	Imaging information	Lumbar degenerative disc changes, lumbar disc herniation, lumbar intervertebral slip, lumbar intervertebral canal stenosis in the relevant segments seen on CT, MRI	Minor improvement and inflammation	Moderate improvement and mild inflammation	Improved and slightly inflamed	Complete improvement and no inflammation
	VAS score	7.34	6.67	5.51	4.80	4.45
75 (Long segment fixation)	Clinical symptom	Pain and numbness in the lower limbs, aggravated by sitting, walking and exertion	Minor improvement and inflammation	Moderate improvement and mild inflammation	Improved and slightly inflamed	Complete improvement and no inflammation
	Disease duration (Years)	14.6	—	—	—	—
	Cobb angle (°)	10.63	8.75	7.08	6.24	5.71
	Imaging information	Lumbar intervertebral slippage and lumbar intervertebral stenosis of the relevant segments seen on CT and MRI	Minor improvement and inflammation	Moderate improvement and mild inflammation	Improved and slightly inflamed	Complete improvement and no inflammation
	VAS score	7.16	6.25	5.45	4.32	4.08
76 (Short segment fixation)	Clinical symptom	Scoliosis deformity with corresponding pressure and percussion pain and radiating neurological symptoms in the lower limbs	Minor improvement and inflammation	Moderate improvement and mild inflammation	Improved and slightly inflamed	Complete improvement and no inflammation
	Disease duration (Years)	15.6	—	—	—	—
	Cobb angle (°)	10.89	7.74	7.02	5.37	4.59
	Imaging information	Lumbar disc herniation, lumbar intervertebral slip, lumbar intervertebral canal stenosis in the relevant segment seen on CT, MRI	Minor improvement and inflammation	Moderate improvement and mild inflammation	Improved and slightly inflamed	Complete improvement and no inflammation
	VAS score	7.48	6.52	5.71	4.93	4.47
77 (Short segment fixation)	Clinical symptom	Pathological reflexes are demonstrated by a straight leg raise test (+)	Minor improvement and inflammation	Moderate improvement and mild inflammation	Improved and slightly inflamed	Complete improvement and no inflammation
		15.1	—	—	—	—

(Continued)

Table S1: *Continued*

Patients		Pre operative	3 months post operative	6 months post operative	12 months post operative	Follow up
	Disease duration (Years)					
	Cobb angle (°)	10.73	8.15	7.28	5.68	4.83
	Imaging information	Lumbar disc herniation and lumbar intervertebral canal stenosis in the relevant segment seen on CT and MRI	Minor improvement and inflammation	Moderate improvement and mild inflammation	Improved and slightly inflamed	Complete improvement and no inflammation
	VAS score	7.45	6.36	5.78	4.92	4.31
78 (Long segment fixation)	Clinical symptom	Soreness and pain in the lower back and legs recurring over a long period of time	Minor improvement and inflammation	Moderate improvement and mild inflammation	Improved and slightly inflamed	Complete improvement and no inflammation
	Disease duration (Years)	16.7	—	—	—	—
	Cobb angle (°)	11.75	9.07	7.36	6.22	5.94
	Imaging information	Degenerative changes of lumbar intervertebral discs and lumbar intervertebral spinal stenosis in the relevant segments were seen on CT and MRI	Minor improvement and inflammation	Moderate improvement and mild inflammation	Improved and slightly inflamed	Complete improvement and no inflammation
	VAS score	7.33	6.57	5.91	4.85	4.28
79 (Short segment fixation)	Clinical symptom	Chronic back pain	Minor improvement and inflammation	Moderate improvement and mild inflammation	Improved and slightly inflamed	Complete improvement and no inflammation
	Disease duration (Years)	10.5	—	—	—	—
	Cobb angle (°)	11.14	8.38	7.29	5.47	4.33
	Imaging information	Degenerative changes of lumbar intervertebral discs and lumbar intervertebral spinal stenosis in the relevant segments were seen on CT and MRI	Minor improvement and inflammation	Moderate improvement and mild inflammation	Improved and slightly inflamed	Complete improvement and no inflammation
	VAS score	7.27	6.19	5.34	4.89	4.22
80 (Short segment fixation)	Clinical symptom	Scoliosis deformity with corresponding pressure and percussion pain and radiating	Minor improvement and inflammation	Moderate improvement and mild inflammation	Improved and slightly inflamed	Complete improvement and no inflammation

(Continued)

Table S1: *Continued*

Patients		Pre operative	3 months post operative	6 months post operative	12 months post operative	Follow up
		neurological symptoms in the lower limbs				
	Disease duration (Years)	9.9	—	—	—	—
	Cobb angle (°)	10.72	7.84	7.26	5.73	4.85
	Imaging information	Lumbar disc herniation and lumbar intervertebral spondylolisthesis of the relevant segments seen on CT and MRI	Minor improvement and inflammation	Moderate improvement and mild inflammation	Improved and slightly inflamed	Complete improvement and no inflammation
	VAS score	7.32	6.47	5.91	4.83	4.25
81 (Long segment fixation)	Clinical symptom	Pain and numbness in the lower limbs, aggravated by sitting, walking and exertion	Minor improvement and inflammation	Moderate improvement and mild inflammation	Improved and slightly inflamed	Complete improvement and no inflammation
	Disease duration (Years)	13.1	—	—	—	—
	Cobb angle (°)	10.84	9.37	7.45	5.72	5.91
	Imaging information	Lumbar intervertebral slippage and lumbar intervertebral stenosis of the relevant segments seen on CT and MRI	Minor improvement and inflammation	Moderate improvement and mild inflammation	Improved and slightly inflamed	Complete improvement and no inflammation
	VAS score	7.32	6.53	5.61	4.78	4.24
82 (Short segment fixation)	Clinical symptom	Soreness and pain in the lower back and legs recurring over a long period of time	Minor improvement and inflammation	Moderate improvement and mild inflammation	Improved and slightly inflamed	Complete improvement and no inflammation
	Disease duration (Years)	9.6	—	—	—	—
	Cobb angle (°)	11.57	8.48	7.15	5.56	4.73
	Imaging information	Lumbar intervertebral slippage and lumbar intervertebral stenosis of the relevant segments seen on CT and MRI	Minor improvement and inflammation	Moderate improvement and mild inflammation	Improved and slightly inflamed	Complete improvement and no inflammation
	VAS score	7.37	6.45	5.92	4.61	4.21
83 (Short segment fixation)	Clinical symptom	Pain and numbness in the lower limbs or difficulty in walking, aggravated by standing or	Minor improvement and inflammation	Moderate improvement and mild inflammation	Improved and slightly inflamed	Complete improvement and no inflammation

(Continued)

Table S1: *Continued*

Patients		Pre operative	3 months post operative	6 months post operative	12 months post operative	Follow up
		walking and relieved by lying down				
	Disease duration (Years)	9.5	—	—	—	—
	Cobb angle (°)	10.91	7.74	7.32	5.45	4.67
	Imaging information	Lumbar disc herniation and lumbar intervertebral canal stenosis in the relevant segment seen on CT and MRI	Minor improvement and inflammation	Moderate improvement and mild inflammation	Improved and slightly inflamed	Complete improvement and no inflammation
	VAS score	7.28	6.45	5.67	4.82	4.43
84 (Long segment fixation)	Clinical symptom	Pain and numbness in the lower limbs, aggravated by sitting, walking and exertion	Minor improvement and inflammation	Moderate improvement and mild inflammation	Improved and slightly inflamed	Complete improvement and no inflammation
	Disease duration (Years)	10.2	—	—	—	—
	Cobb angle (°)	10.97	9.85	8.26	7.08	6.42
	Imaging information	Lumbar disc herniation, lumbar intervertebral slip, lumbar intervertebral canal stenosis in the relevant segment seen on CT, MRI	Minor improvement and inflammation	Moderate improvement and mild inflammation	Improved and slightly inflamed	Complete improvement and no inflammation
	VAS score	7.28	6.41	5.35	4.46	4.23
85 (Short segment fixation)	Clinical symptom	Soreness and pain in the lower back and legs recurring over a long period of time	Minor improvement and inflammation	Moderate improvement and mild inflammation	Improved and slightly inflamed	Complete improvement and no inflammation
	Disease duration (Years)	13.1	—	—	—	—
	Cobb angle (°)	10.73	7.38	6.41	5.29	4.67
	Imaging information	Degenerative changes of lumbar intervertebral discs in the relevant segments, lumbar intervertebral disc herniation visible under CT, MRI	Minor improvement and inflammation	Moderate improvement and mild inflammation	Improved and slightly inflamed	Complete improvement and no inflammation
	VAS score	7.56	6.73	5.92	4.97	4.45
		Presence of trauma				

(Continued)

Table S1: *Continued*

Patients		Pre operative	3 months post operative	6 months post operative	12 months post operative	Follow up
86 (Short segment fixation)	Clinical symptom		Minor improvement and inflammation	Moderate improvement and mild inflammation	Improved and slightly inflamed	Complete improvement and no inflammation
	Disease duration (Years)	13.4	—	—	—	—
	Cobb angle (°)	11.47	8.53	6.94	5.68	4.26
	Imaging information	Lumbar degenerative disc changes, lumbar disc herniation, lumbar intervertebral slip, lumbar intervertebral canal stenosis in the relevant segments seen on CT, MRI	Minor improvement and inflammation	Moderate improvement and mild inflammation	Improved and slightly inflamed	Complete improvement and no inflammation
	VAS score	7.27	6.21	5.58	4.74	4.20
87 (Long segment fixation)	Clinical symptom	Chronic back pain	Minor improvement and inflammation	Moderate improvement and mild inflammation	Improved and slightly inflamed	Complete improvement and no inflammation
	Disease duration (Years)	13.6	—	—	—	—
	Cobb angle (°)	12.63	11.79	9.11	7.25	6.38
	Imaging information	Lumbar disc herniation and lumbar intervertebral canal stenosis in the relevant segment seen on CT and MRI	Minor improvement and inflammation	Moderate improvement and mild inflammation	Improved and slightly inflamed	Complete improvement and no inflammation
	VAS score	7.26	6.45	5.91	4.77	4.34
88 (Short segment fixation)	Clinical symptom	Intermittent claudication with marked neurogenic symptoms	Minor improvement and inflammation	Moderate improvement and mild inflammation	Improved and slightly inflamed	Complete improvement and no inflammation
	Disease duration (Years)	14.2	—	—	—	—
	Cobb angle (°)	10.74	7.36	6.45	5.21	4.49
	Imaging information	Lumbar intervertebral slippage and lumbar intervertebral stenosis of the relevant segments seen on CT and MRI	Minor improvement and inflammation	Moderate improvement and mild inflammation	Improved and slightly inflamed	Complete improvement and no inflammation
	VAS score	7.19	6.58	5.91	4.87	4.14

(Continued)

Table S1: *Continued*

Patients		Pre operative	3 months post operative	6 months post operative	12 months post operative	Follow up
89 (Short segment fixation)	Clinical symptom	Scoliosis deformity with corresponding pressure and percussion pain and radiating neurological symptoms in the lower limbs	Minor improvement and inflammation	Moderate improvement and mild inflammation	Improved and slightly inflamed	Complete improvement and no inflammation
	Disease duration (Years)	13.4	—	—	—	—
	Cobb angle (°)	10.65	7.45	6.36	5.58	3.91
	Imaging information	Radiographs suggest a scoliotic deformity with a Cobb >10°, which may be associated with slippage or stenosis	Minor improvement and inflammation	Moderate improvement and mild inflammation	Improved and slightly inflamed	Complete improvement and no inflammation
	VAS score	7.43	5.79	4.68	3.51	2.72
90 (Long segment fixation)	Clinical symptom	Pathological reflexes are demonstrated by a straight leg raise test (+)	Minor improvement and inflammation	Moderate improvement and mild inflammation	Improved and slightly inflamed	Complete improvement and no inflammation
	Disease duration (Years)	12.9	—	—	—	—
	Cobb angle (°)	10.47	9.06	7.84	6.52	6.35
	Imaging information	Degenerative changes of lumbar intervertebral discs in the relevant segments, lumbar intervertebral spondylolisthesis, as seen on CT, MRI	Minor improvement and inflammation	Moderate improvement and mild inflammation	Improved and slightly inflamed	Complete improvement and no inflammation
	VAS score	7.29	5.35	4.37	3.78	3.28
91 (Short segment fixation)	Clinical symptom	Soreness and pain in the lower back and legs recurring over a long period of time	Minor improvement and inflammation	Moderate improvement and mild inflammation	Improved and slightly inflamed	Complete improvement and no inflammation
	Disease duration (Years)	11.5	—	—	—	—
	Cobb angle (°)	11.27	8.64	7.12	5.93	4.15
	Imaging information	Lumbar disc herniation, lumbar intervertebral slip, lumbar intervertebral canal stenosis in	Minor improvement and inflammation	Moderate improvement and mild inflammation	Improved and slightly inflamed	Complete improvement and no inflammation

(Continued)

Table S1: *Continued*

Patients		Pre operative	3 months post operative	6 months post operative	12 months post operative	Follow up
		the relevant segment seen on CT, MRI				
	VAS score	7.49	5.57	4.28	3.51	3.06
92 (Short segment fixation)	Clinical symptom	Chronic back pain	Minor improvement and inflammation	Moderate improvement and mild inflammation	Improved and slightly inflamed	Complete improvement and no inflammation
	Disease duration (Years)	12.1	—	—	—	—
	Cobb angle (°)	11.48	8.57	7.25	5.56	4.72
	Imaging information	Lumbar degenerative disc changes, lumbar disc herniation, lumbar intervertebral slip, lumbar intervertebral canal stenosis in the relevant segments seen on CT, MRI	Minor improvement and inflammation	Moderate improvement and mild inflammation	Improved and slightly inflamed	Complete improvement and no inflammation
	VAS score	7.52	5.78	4.41	3.74	3.13
93 (Long segment fixation)	Clinical symptom	Chronic back pain	Minor improvement and inflammation	Moderate improvement and mild inflammation	Improved and slightly inflamed	Complete improvement and no inflammation
	Disease duration (Years)	12.4	—	—	—	—
	Cobb angle (°)	10.92	9.35	7.46	6.73	5.91
	Imaging information	Degenerative changes of lumbar intervertebral discs and lumbar intervertebral spinal stenosis in the relevant segments were seen on CT and MRI	Minor improvement and inflammation	Moderate improvement and mild inflammation	Improved and slightly inflamed	Complete improvement and no inflammation
	VAS score	7.36	5.27	3.79	2.91	2.52
94 (Short segment fixation)	Clinical symptom	Soreness and pain in the lower back and legs recurring over a long period of time	Minor improvement and inflammation	Moderate improvement and mild inflammation	Improved and slightly inflamed	Complete improvement and no inflammation
	Disease duration (Years)	13.6	—	—	—	—
	Cobb angle (°)	10.74	7.59	7.21	5.49	4.43

(Continued)

Table S1: *Continued*

Patients		Pre operative	3 months post operative	6 months post operative	12 months post operative	Follow up
	Imaging information	Lumbar intervertebral slippage and lumbar intervertebral stenosis of the relevant segments seen on CT and MRI	Minor improvement and inflammation	Moderate improvement and mild inflammation	Improved and slightly inflamed	Complete improvement and no inflammation
	VAS score	7.13	5.45	4.26	3.57	3.24
95 (Short segment fixation)	Clinical symptom	Intermittent claudication with marked neurogenic symptoms	Minor improvement and inflammation	Moderate improvement and mild inflammation	Improved and slightly inflamed	Complete improvement and no inflammation
	Disease duration (Years)	14.7	—	—	—	—
	Cobb angle (°)	10.82	8.13	7.38	5.07	3.64
	Imaging information	Lumbar disc herniation and lumbar intervertebral canal stenosis in the relevant segment seen on CT and MRI	Minor improvement and inflammation	Moderate improvement and mild inflammation	Improved and slightly inflamed	Complete improvement and no inflammation
	VAS score	7.13	6.22	5.02	4.25	3.84
96 (Long segment fixation)	Clinical symptom	Pain and numbness in the lower limbs, aggravated by sitting, walking and exertion	Minor improvement and inflammation	Moderate improvement and mild inflammation	Improved and slightly inflamed	Complete improvement and no inflammation
	Disease duration (Years)	13.7	—	—	—	—
	Cobb angle (°)	11.46	10.58	8.21	6.45	5.75
	Imaging information	Lumbar intervertebral slippage and lumbar intervertebral stenosis of the relevant segments seen on CT and MRI	Minor improvement and inflammation	Moderate improvement and mild inflammation	Improved and slightly inflamed	Complete improvement and no inflammation
	VAS score	7.48	5.62	4.17	3.25	2.95

Table S2: MRI (T2W1) grading criteria for Pfirrmann's disc degeneration

Classification	Structure	Nucleus pulposus and fibrous ring border	Signal	Intervertebral disc height
I	Even texture, bright white colour	Clear	High or equal to cerebrospinal fluid	Normal
II	Non-homogeneous, with or without horizontal bands	Clear	High or equal to cerebrospinal fluid	Normal
III	Non-homogeneous, grey	Unclear	Moderate	Normal or mildly reduced
IV	Non-homogeneous, grey or black	Disappear	Medium or low signal	Normal or moderately reduced
V	Non-homogeneous, black	Disappear	Low Signal	Collapse of the intervertebral disc space

(a)

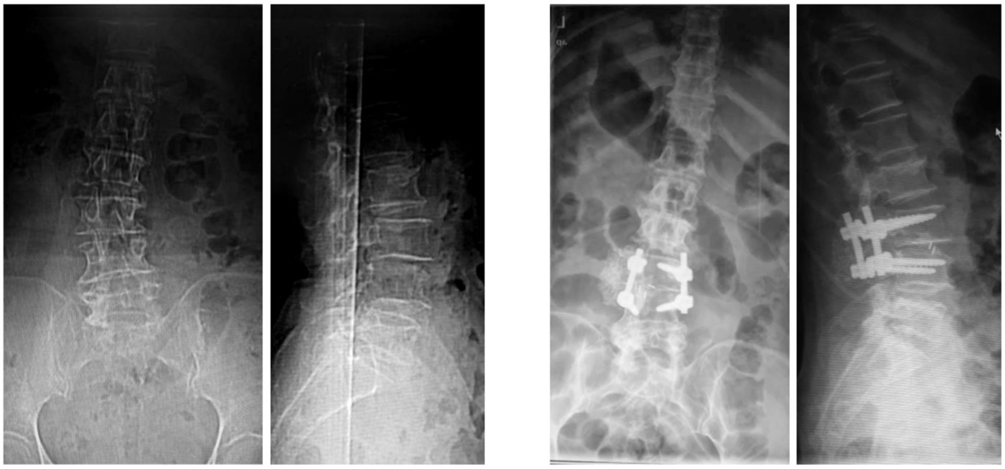

(b)

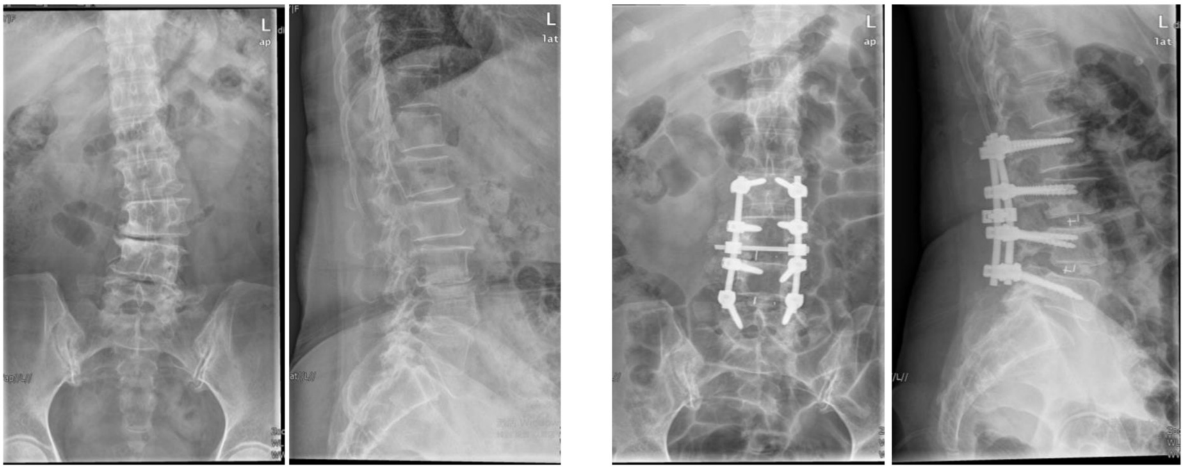

Figure S1: Pre-operative and post-operative imaging comparisons between patients undergoing short-segment and long-segment fixations. (a) Pre-operative (left) and post-operative (right) imaging of a patient with short segments; (b) pre-operative (left) and post-operative (right) imaging of a patient with long segments.
